# MISpheroID: a knowledgebase and transparency tool for minimum information in spheroid identity

**DOI:** 10.1038/s41592-021-01291-4

**Published:** 2021-11-01

**Authors:** Arne Peirsman, Eva Blondeel, Tasdiq Ahmed, Jasper Anckaert, Dominique Audenaert, Tom Boterberg, Krisztina Buzas, Neil Carragher, Gastone Castellani, Flávia Castro, Virginie Dangles-Marie, John Dawson, Pascal De Tullio, Elly De Vlieghere, Sándor Dedeyne, Herman Depypere, Akos Diosdi, Ruslan I. Dmitriev, Helmut Dolznig, Suzanne Fischer, Christian Gespach, Vera Goossens, Jyrki Heino, An Hendrix, Peter Horvath, Leoni A. Kunz-Schughart, Sebastiaan Maes, Christophe Mangodt, Pieter Mestdagh, Soňa Michlíková, Maria José Oliveira, Francesco Pampaloni, Filippo Piccinini, Cláudio Pinheiro, Jennifer Rahn, Stephen M. Robbins, Elina Siljamäki, Patrick Steigemann, Gwen Sys, Shuichi Takayama, Anna Tesei, Joeri Tulkens, Michiel Van Waeyenberge, Jo Vandesompele, Glenn Wagemans, Claudia Weindorfer, Nurten Yigit, Nina Zablowsky, Michele Zanoni, Phillip Blondeel, Olivier De Wever

**Affiliations:** 1 Laboratory of Experimental Cancer Research, Cancer Research Institute, Ghent, Belgium; 2 Department of Human Structure and Repair, Ghent University, Ghent, Belgium; 3 Plastic, Reconstructive and Aesthetic Surgery, Ghent University Hospital, Ghent, Belgium; 4 Wallace H Coulter Department of Biomedical Engineering and Petit Institute for Bioengineering and Bioscience, Georgia Institute of Technology and Emory School of Medicine, Atlanta, GA USA; 5 OncoRNALab, Cancer Research Institute, Ghent, Belgium; 6 Department of Biomolecular Medicine, Ghent University, Ghent, Belgium; 7 VIB Screening Core and Ghent University Expertise Centre for Bioassay Development and Screening (C-BIOS-VIB), Ghent University, Ghent, Belgium; 8 Department of Radiation Oncology, Ghent University Hospital, Ghent, Belgium; 9 Department of Immunology, University of Szeged, Faculty of Medicine-Faculty of Science and Informatics, Szeged, Hungary; 10 Institute of Genetics and Cancer, Cancer Research UK Edinburgh Centre, University of Edinburgh, Edinburgh, UK; 11 Department of Experimental, Diagnostic and Specialty Medicine, University of Bologna, Bologna, Italy; 12 i3S – Institute for Research and Innovation in Health, University of Porto, Porto, Portugal; 13 Translational Research Department, Institut Curie, PSL Research University, and Faculty of Pharmacy, Paris, France; 14 Faculty of Pharmacy, Université Paris Descartes, Paris, France; 15 Center for Interdisciplinary Research on Medicines (CIRM), Metabolomics Group, Université de Liège, Liège, Belgium; 16 Menopause and Breast Clinic, Ghent University Hospital, Ghent, Belgium; 17 Synthetic and Systems Biology Unit, Hungarian Academy of Sciences, Biological Research Center (BRC), Szeged, Hungary; 18 Tissue Engineering and Biomaterials Group, Department of Human Structure and Repair, Ghent University, Ghent, Belgium; 19 Institute of Medical Genetics, Medical University of Vienna, Vienna, Austria; 20 INSERM U938 Hospital Saint-Antoine Research Center CRSA, Team Céline Prunier, TGFbeta Signaling in Cellular Plasticity and Cancer, Sorbonne University, Paris, France; 21 Department of Life Technologies, University of Turku, Turku, Finland; 22 OncoRay – National Center for Radiation Research in Oncology, University Hospital Carl Gustav Carus Dresden, Carl Gustav Carus Faculty of Medicine at TU Dresden, and Helmholtz-Zentrum Dresden–Rossendorf, Dresden, Germany; 23 Physical Biology Group, Buchmann Institute for Molecular Life Sciences (BMLS), Goethe Universität Frankfurt am Main, Frankfurt am Main, Germany; 24 IRCCS Istituto Romagnolo per lo Studio dei Tumori (IRST) ‘Dino Amadori’, Meldola, Italy; 25 Departments of Oncology and Biochemistry and Molecular Biology, Cumming School of Medicine, University of Calgary, Calgary, Alberta Canada; 26 Lead Discovery, Nuvisan ICB, Berlin, Germany; 27 Department of Orthopedics and Traumatology, Ghent University Hospital, Ghent University, Ghent, Belgium

**Keywords:** Research data, Cell biology, Biological models

## Abstract

Spheroids are three-dimensional cellular models with widespread basic and translational application across academia and industry. However, methodological transparency and guidelines for spheroid research have not yet been established. The MISpheroID Consortium developed a crowdsourcing knowledgebase that assembles the experimental parameters of 3,058 published spheroid-related experiments. Interrogation of this knowledgebase identified heterogeneity in the methodological setup of spheroids. Empirical evaluation and interlaboratory validation of selected variations in spheroid methodology revealed diverse impacts on spheroid metrics. To facilitate interpretation, stimulate transparency and increase awareness, the Consortium defines the MISpheroID string, a minimum set of experimental parameters required to report spheroid research. Thus, MISpheroID combines a valuable resource and a tool for three-dimensional cellular models to mine experimental parameters and to improve reproducibility.

## Abstract (web summary)

A knowledgebase developed for increased the transparency of reporting in spheroid research.

## Main

Spheroids, which are near-spherical multicellular aggregates, are one of the most common types of three-dimensional (3D) cell cultures. In contrast to two-dimensional (2D) cell cultures, spheroids have the advantage of maintaining a diffusive nutrient and oxygen supply, leading to metabolic gradients from the periphery to the inner core and causing spatial heterogeneity in proliferation, quiescence, necrosis and differentiation^1,2^. Spheroids are used as simplified biomimetic in vitro models to study fundamental mechanisms in biology and can be generated from a variety of cell cultures from healthy as well as pathological tissue, including cancer. Their scalability has promoted academic and industrial interest, particularly in the evaluation of drug responses or the biofabrication of 3D functional tissues or organs^3–8^. The scientific literature uses several alternative terms, such as spheres, tumor(o)spheres and mammospheres, with each having a different definition^9^. To avoid confusion, the general term ‘spheroid’ will be used, to cover all aforementioned terms.

Spheroid production is based on the principle of self-assembly, which occurs when cells present in a non-adherent environment aggregate together. To induce these cellular interactions, numerous spheroid formation methods have been established such as spinner flasks, hanging drop cultures, microfluidic devices, cultures on low-adhesive substrates and so on^3^. Spheroid biology, including cellular interactions and cell death, severely affect drug responsiveness^10,11^. Specific changes in methodological setup, such as the nutrient composition of cell culture media and the choice of spheroid formation method, may also contribute to differences in spheroid metrics^12–16^. Although there is no one-size-fits-all methodological setup for spheroid experiments, it is currently unclear how heterogeneity in methodology affects spheroid metrics. Consequently, the usage of a diverse set of experimental settings requires transparent reporting, without which results are difficult to interpret, compare and reproduce^17,18^.

Despite decades of implementation of spheroid technology in various fields of life science and medical research^19,20^, no minimum information (MI) guidelines are available to cope with heterogeneity and encourage transparency. Minimum Information for Biological and Biomedical Investigations (MIBBI) provides access to the Minimum Information About a Cellular Assay (MIACA) and Minimum Information About Cell Migration Experiments (MIACME) guidelines, but these resources are not specific nor sufficient for spheroid experimentation given that they do not cope with the complexity of a 3D experiment^21–23^. This situation is in contrast to that for other biological fields, in which minimum information initiatives are available to define field-specific biological and technical parameters^24–26^.

To cope with this unmet need, we assembled an international consortium to develop the MISpheroID knowledgebase (https://www.mispheroid.org). In-depth empirical evaluation and interlaboratory validation of selected variations in methodological setup identified a significant impact on a diverse set of spheroid metrics, while interrogation identified heterogeneity and a lack of transparency in published spheroid-related experiments. These results are merged into the generation of a minimum information string for spheroid interpretation. Thus, MISpheroID is a unique open-access resource that facilitates systematic reporting on essential spheroid methodology with the aim to increase consistency and awareness in both academic and industrial research environments.

## Results

### Creation of the MISpheroID knowledgebase

An initial literature screening identified spheroids derived from breast cancer cells as the most reported in the past decade (Supplementary Table 1). Consequently, we first conducted an in-depth methodological analysis of 1,628 breast cancer spheroid-related experiments, of which 1,506 were of human and 122 were of animal origin (Supplementary Fig. 1). For each experiment, we completed a checklist of 98 parameters relating to spheroid setup, characterization and application (Supplementary Table 2). Next, spheroid-related experiments from other tumor sites including the brain (*n* = 248), colorectum (*n* = 324), liver (*n* = 211), lung (*n* = 213), ovary (*n* = 243) and pancreas (*n* = 191) were evaluated, which resulted in an additional set of 1,430 experiments. Data were curated before inclusion in the MISpheroID knowledgebase, which to date includes a total of 3,058 experiment entries.

### Spheroid research practices

**Fig. 1:**
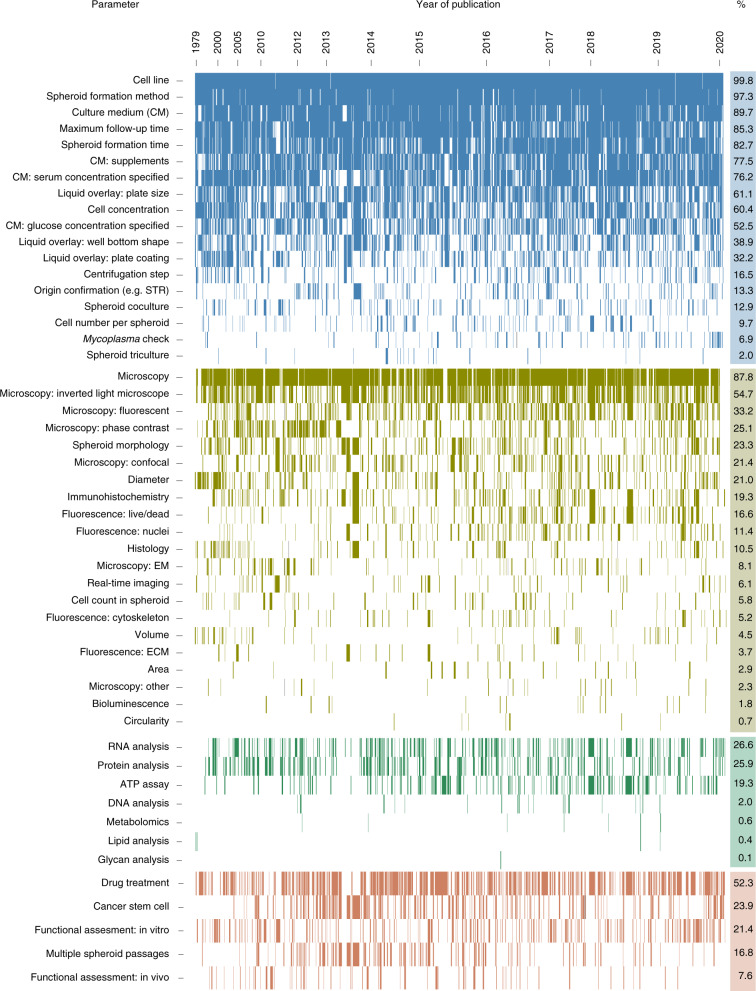
Mapping the reporting topography in breast cancer spheroid research. Binary heatmap showing the experimental parameters (rows, 51 of 98 parameters, selected for relevance) of each spheroid experiment (columns, *n* = 1,628). The heatmap is divided vertically into three sections of parameters (‘setup’, ‘characterization both microscopic and non-microscopic’ and ‘application’; indicated in blue, light and dark green, and red; and including 18, 21 + 7, and 5 parameters, respectively) and horizontally according to the year of publication. For each section, rows are sorted in descending order according to total number of reported experimental parameters. Parameters that were not reported in an experiment appear as a white space in its corresponding column. The reporting efficiency of each parameter is indicated as a percentage in the right column. EM, electron microscopy; ECM, extracellular matrix. Source data

**Extended Data Fig. 1:**
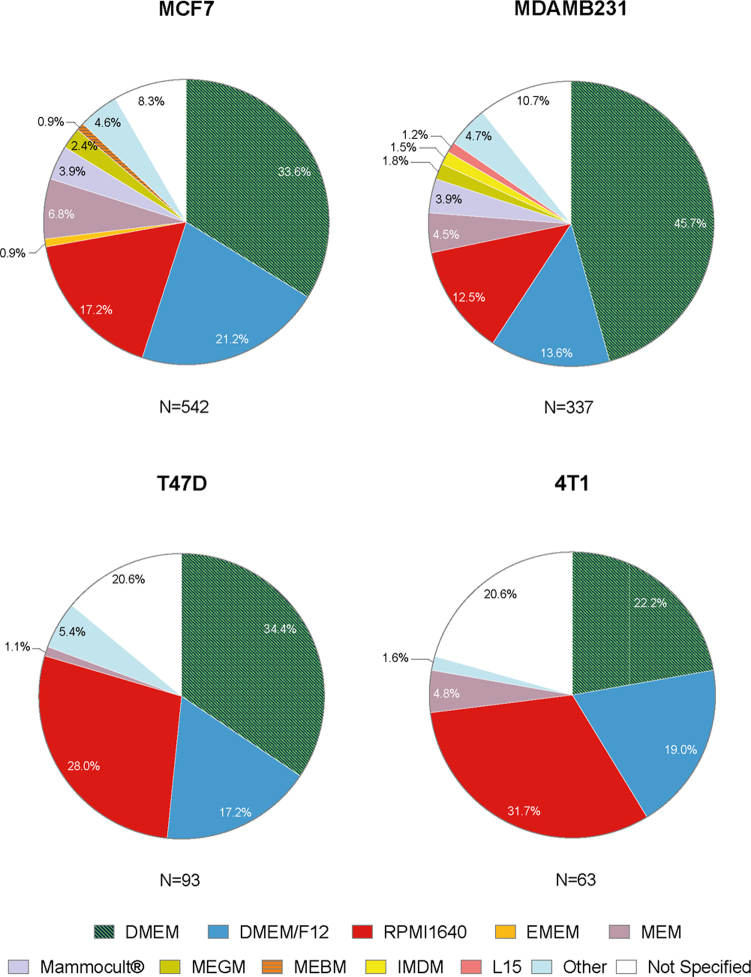
Distribution of the applied culture medium to prepare spheroids of the estrogen-dependent MCF7 and T47D, and triple-negative MDAMB231 and 4T1 breast/mammary gland cancer cell lines. Pie chart visualizing the proportion of culture media used to prepare MCF7, T47D, MDAMB231 and 4T1 spheroids. Source data

To identify practices in spheroid research, we performed an in-depth analysis of the MISpheroID knowledgebase. This found that 1,333 (of 1628, 81.9%) unique protocols have been reported to establish and characterize breast cancer spheroids (Supplementary Fig. 2). Visualization of MISpheroID data shows inconsistent reporting and/or heterogeneity in breast cancer spheroid setup, characterization and application (Fig. 1). Culture medium type is not reported in 10% of experiments. In 47.5% of experiments the glucose concentration is not disclosed. This discrepancy probably results from the availability of 5.6 mM (low glucose, LG) and 25 mM (high glucose, HG) glucose-containing Dulbecco’s Modified Eagle’s Medium (DMEM) formulations and the ambiguous use of the term ‘DMEM’ to include all formulations of the medium. The spheroid formation method is efficiently reported (97.3%), with liquid overlay as the most frequently applied method (71.1%, Supplementary Fig. 3). Of these experiments, 52% use ultra-low attachment (ULA) plates and 42.1% use in situ coated plates. Agarose and poly-HEMA (46.9% and 28.8%, respectively) are the most implemented in situ coats. MISpheroID identifies 79 unique breast or mammary gland cell lines, with the estrogen-dependent MCF7 and T47D (542 (33.3%) and 96 (5.7%) out of 1,628 experiments, respectively) and the triple-negative MDAMB231 and 4T1 (337 (20.7%) and 63 (3.8%) out of 1,628 experiments, respectively) as the most frequently used breast or mammary gland cancer cell lines (Supplementary Fig. 4 and Supplementary Table 3). For each of these cell lines, a diverse set of culture media and formation methods is used to establish and study spheroids, although both variables are known to affect spheroid metrics^11,12,14–16,27–30^ (Extended Data Fig. 1 and Supplementary Fig. 5).

Although 87.8% of the breast cancer spheroid experiments are characterized by microscopy-based techniques, only 23.3% provide information about spheroid morphology. Despite the potential influence of spheroid size on study conclusions^10,11^, the numerical reporting of spheroid diameter (size), volume and projected area is described only in 21.0%, 4.5% and 2.9% of experiments, respectively. Shape assessment (for example, circularity) is performed in less than 1% of experiments. Characterization by non-microscopy-based techniques is mainly focused on RNA (for example, quantitative polymerase chain reaction, qPCR) and protein (for example, western blot) analysis in 26.6% and 25.9% of experiments, respectively (Fig. 1).

Spheroid application methods show that 23.9% of experiments focus on cancer stem cells, given that spheroid cultures from specific cell lines can be applied for cancer stem cell enrichment^14^. Functional assessments, such as migration and matrix invasion, are applied in 21.4% of experiments. Studies focused on pathophysiology research and drug testing (48.3% and 24.6%) vastly outnumber those on spheroid 3D culture optimization (18.4%) (Supplementary Fig. 6).

**Fig. 2:**
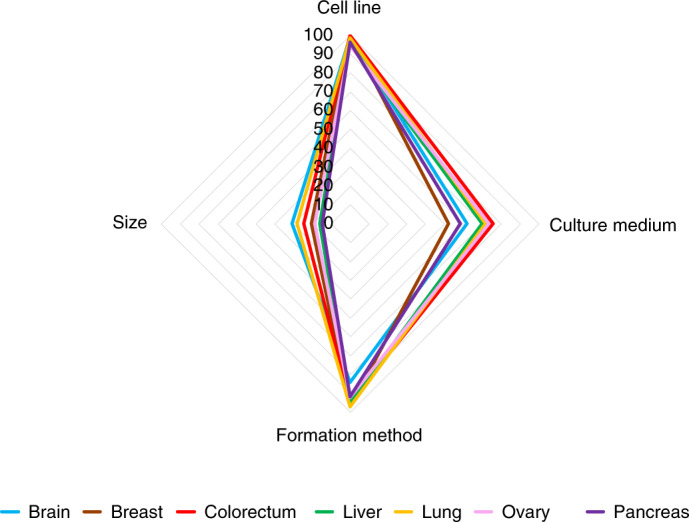
Reporting efficiency of experimental parameters in spheroids from different tumor types. Spider plot visualization of the reporting percentage of cell line, culture medium, formation method and size in spheroids from different tumor types (clockwise). Axes represent the percentage of reporting efficiency. Source data

**Extended Data Fig. 2:**
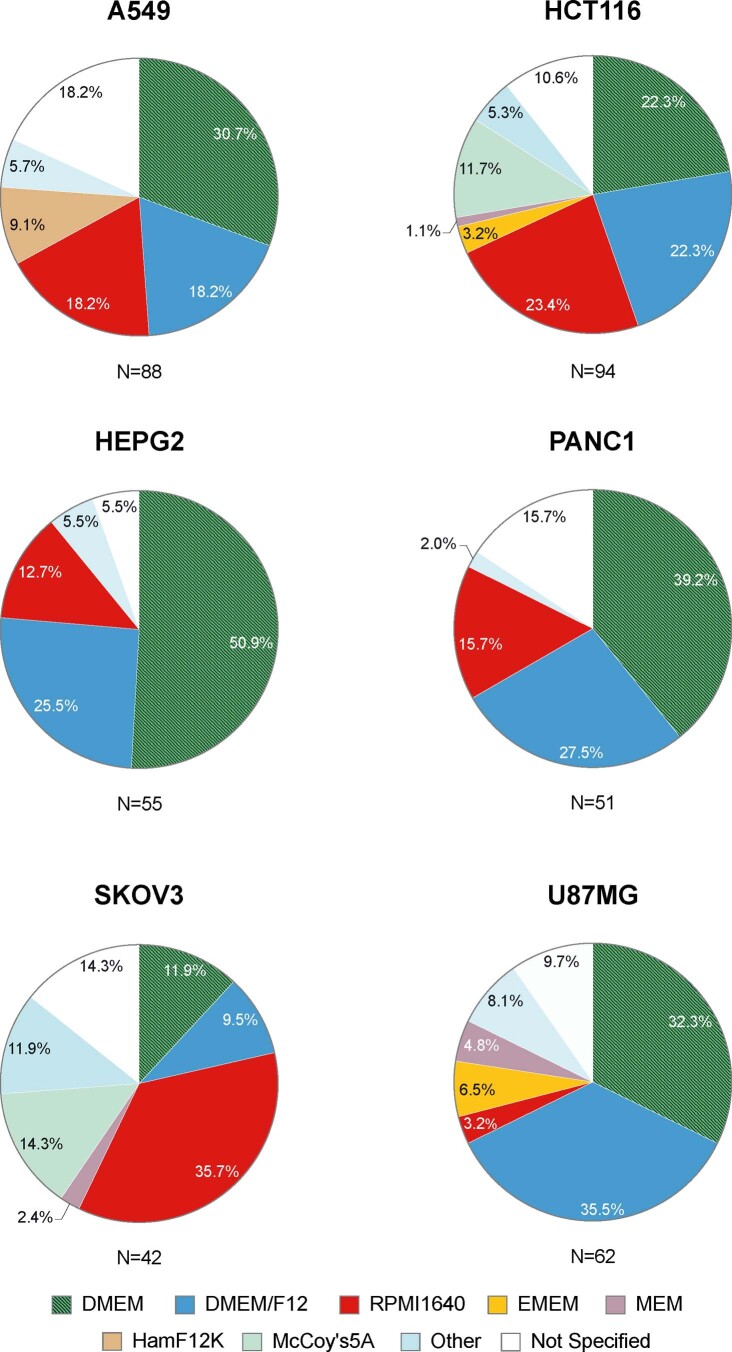
Distribution of the applied culture medium to prepare spheroids of the most reported cell line from non-breast tumors. Pie chart visualizing the proportion of culture media used to prepare A549 (lung), HCT116 (colorectal), HEPG2 (liver), PANC1 (pancreas), SKOV3 (ovarium) and U87MG (brain). Source data

In summary, breast or mammary gland cancer spheroid practices involve heterogeneity and/or lack of reporting in culture medium, spheroid formation method and spheroid size. Evaluation of these parameters for spheroid experiments from other tumor types indicates that lack of reporting in culture medium and spheroid size is widespread in the spheroid research field (Fig. 2). Critically, detailed evaluation of the reported medium types and spheroid formation method for the most frequently used cell line from each tumor type reveals an extensive heterogeneity (Extended Data Fig. 2, Supplementary Figs. 7 and 8 and Supplementary Table 3).

### Impact of methodological heterogeneity on spheroid metrics

The heterogeneity and reporting deficiencies exposed by MISpheroID interrogation prompted us to empirically evaluate the impact of culture medium type, spheroid formation method and spheroid size on complementary spheroid metrics using established cell lines and early passage patient-derived cell cultures from different tumor types.

**Fig. 3:**
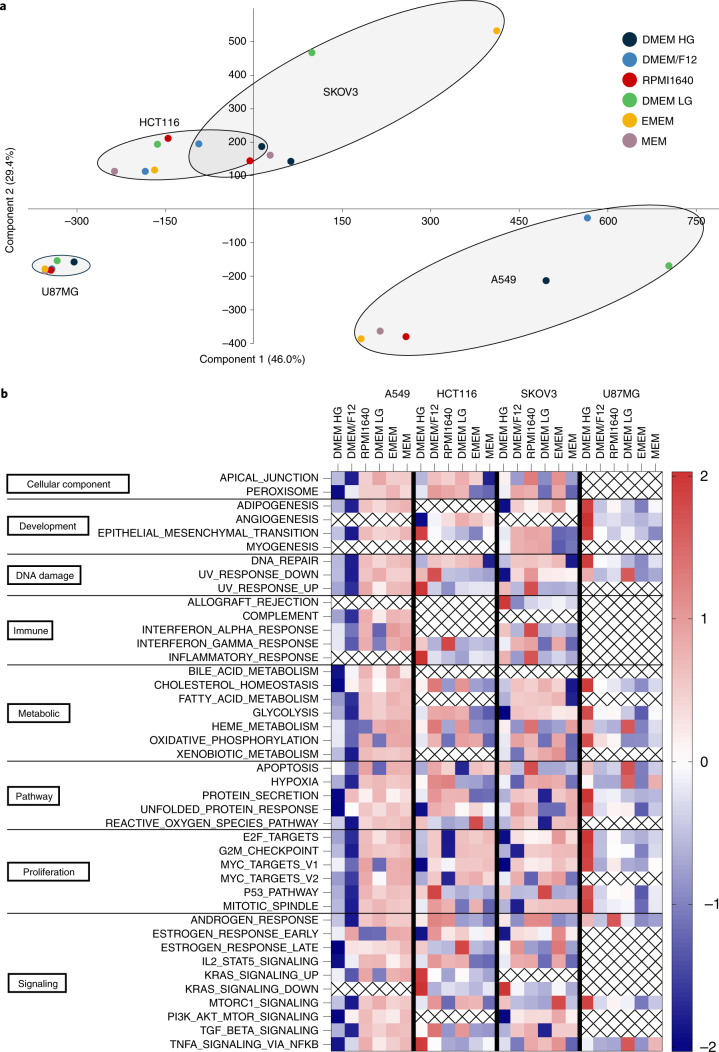
Culture media-induced transcriptional variation. **a**, Principal component analysis of gene expression profiles from spheroids of four cancer cell lines cultured in six different medium types. **b**, Heatmap of Z-scores of all MSigDB hallmark gene sets identified by GSEA to be significantly enriched among the differentially expressed genes across the culture medium types in each cell line. Medium types are ranked from higher nutrient (left) to lower nutrient (right) richness. Source data

**Extended Data Fig. 3:**
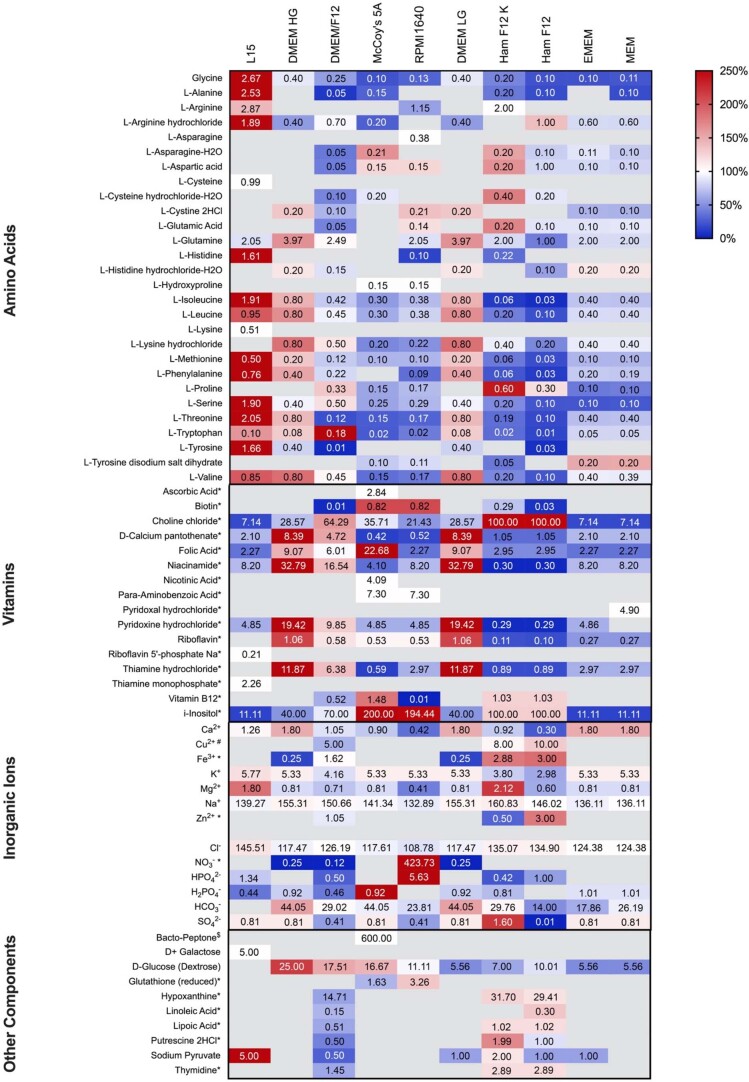
Comparison of the formulation of frequently reported culture media in spheroid research. Heat map showing the formulation of culture media types used in the empirical MISpheroID study, in addition with other media types frequently reported in spheroid research practices including L15, HamF12, HamF12K and McCoy’s5A. Ranked according to decreasing nutrient richness from left to right. Nutrients that are not included in a medium type appear as a grey space in its corresponding column. Left column indicates nutrient type. Numerical value of nutrients in each medium is indicated in each cell. Values are concentrations, expressed in mM, unless indicated *=µM, # =nM, $=mg/l.

Culture medium types commonly reported in the MISpheroID knowledgebase are DMEM (27.3%), DMEM/F12 (25.6%), RPMI1640 (18.1%), Minimum Essential Medium (MEM) (3.5%) and Eagle’s Minimum Essential Medium (EMEM) (0.6%), with each medium type having a different nutrient formulation (Extended Data Fig. 3). We measured media-induced transcriptional variation in lung cancer (A549), colorectal cancer (HCT116), ovarian cancer (SKOV3) and glioblastoma (U87MG) spheroids using RNA sequencing (RNA-seq). Principal component analysis showed differential variation in transcriptional response to culture media, with A549 and SKOV3 having the largest variation (Fig. 3a). For A549 the media separated into two clusters (RPMI1640, EMEM and MEM versus DMEM/F12, DMEM LG and DMEM HG), while for U87MG the variation was less striking but was still apparent, with DMEM HG and DMEM LG in one cluster and the other medium types in a second cluster.

Gene set enrichment analysis (GSEA) using the 50 hallmark gene sets^27^ showed that the differentially expressed genes significantly converged on important hallmarks, and had medium-specific and cell type-specific patterns (Fig. 3b). In agreement with the principal component analyses, A549 and SKOV3 had the largest number of significantly enriched hallmark gene sets (37 and 38 of the 50 analyzed, respectively). The hallmark gene set analysis revealed a unique distinction between medium types within each cell type. While some medium types in the A549 analysis (EMEM and MEM) and in the U87MG analysis (DMEM HG) showed an enrichment of all hallmark signatures, other conditions showed discrete and unique diversities. The largest difference in the enrichment of hallmark gene sets between two medium types was observed for A549, DMEM/F12 versus MEM (mean difference, 1.90), for HCT116, DMEM/F12 versus MEM (mean difference, 1.08), for SKOV3, RPMI1640 versus MEM (mean difference, 1.09), and for U87MG, DMEM HG versus EMEM (mean difference, 2.45) (Supplementary Table 4).

**Fig. 4:**
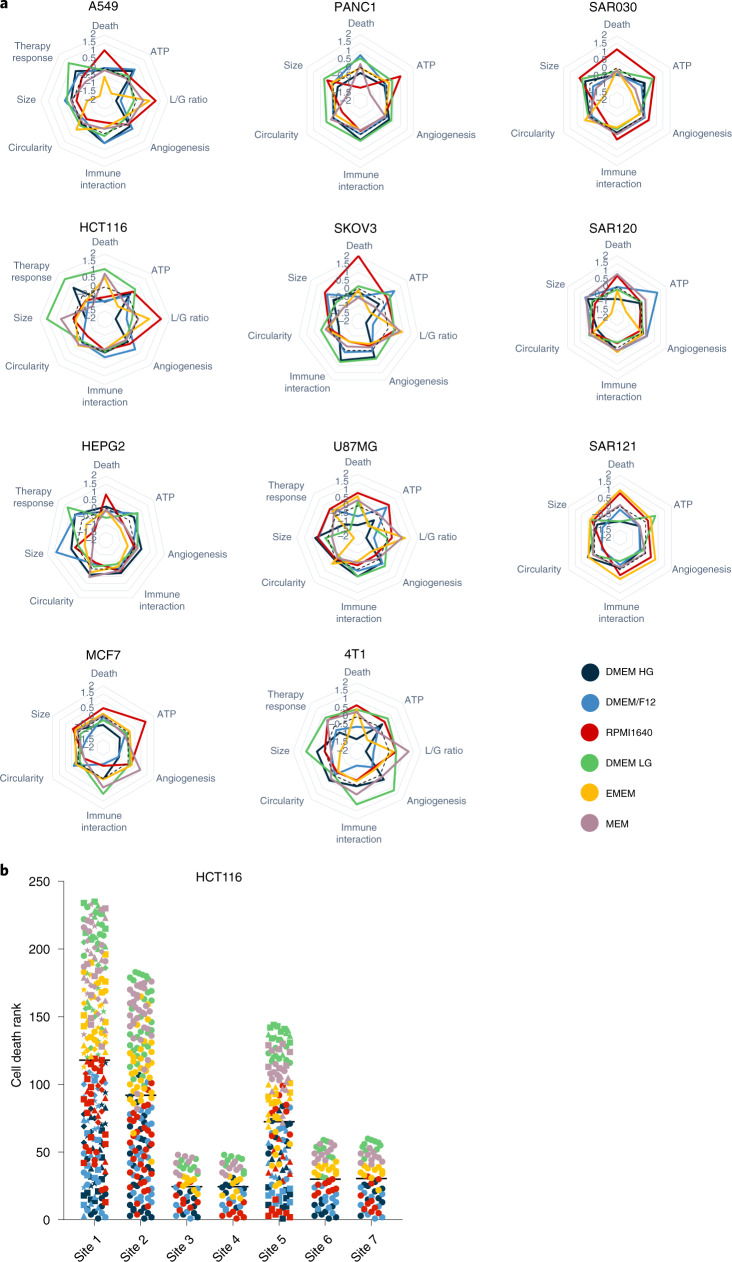
Culture media-induced heterogeneity in spheroid metrics across multiple cell types. **a**, Spider plots of metrics from spheroids of indicated cell lines cultured in six different medium types. Axes represent the Z-score metrics of cell death, ATP content, L/G ratio, secreted protein signatures of angiogenesis and immune interaction, circularity, size and therapy response. A higher Z-score means a higher metric value. The left and middle columns indicate established cell lines; early passage and patient-derived sarcoma cultures are on the right. **b**, Cell death of HCT116 spheroids cultured in six different medium types evaluated at seven different laboratories (“sites”) in an interlaboratory experiment. At each site a higher ranking indicates a higher cell death. Each dot represents an evaluated spheroid. The colors indicate the medium type as in **a**. Source data

**Extended Data Fig. 4:**
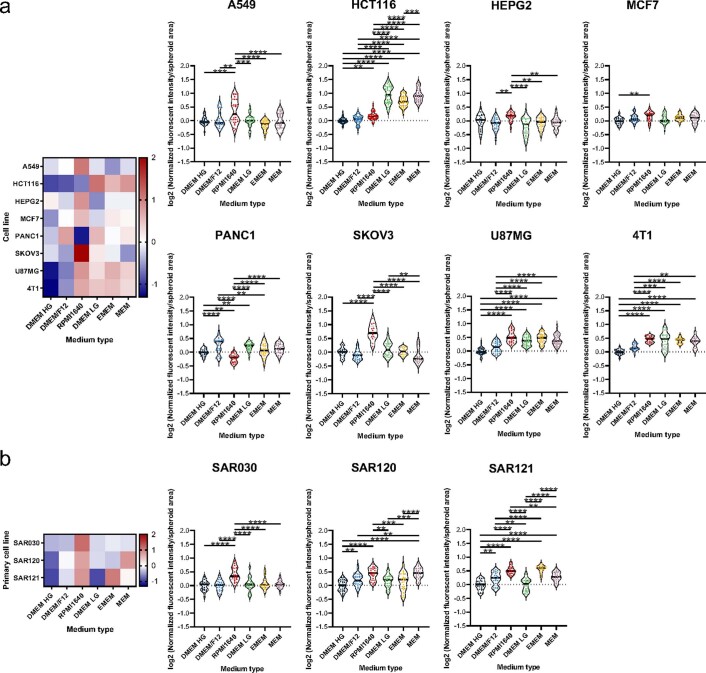
Quantitative presentation of the impact of heterogeneity in culture medium on cell death in spheroids. Left, Z-score heatmaps and right, violin plots presenting the impact of six different media types on cell death in (a) 8 established cell lines and (b) 3 early passage, patient-derived sarcoma cultures. Biological replicates are indicated by a different symbol (N ≥ 3); each symbol is a technical replicate (n = 8). Y-axis represents log2-transformed data, all media types are normalized to DMEM HG. Horizontal bar indicates median. Statistical significance between the groups was determined with a one-way ANOVA and Tukey’s multiple comparison test. **p < 0.01, ***p < 0.001, ****p < 0.0001. Colors in violin plots present media type as in Fig. 3a; media types are ranked from higher nutrient (left) to lower nutrient (right) richness. Source data

**Extended Data Fig. 5:**
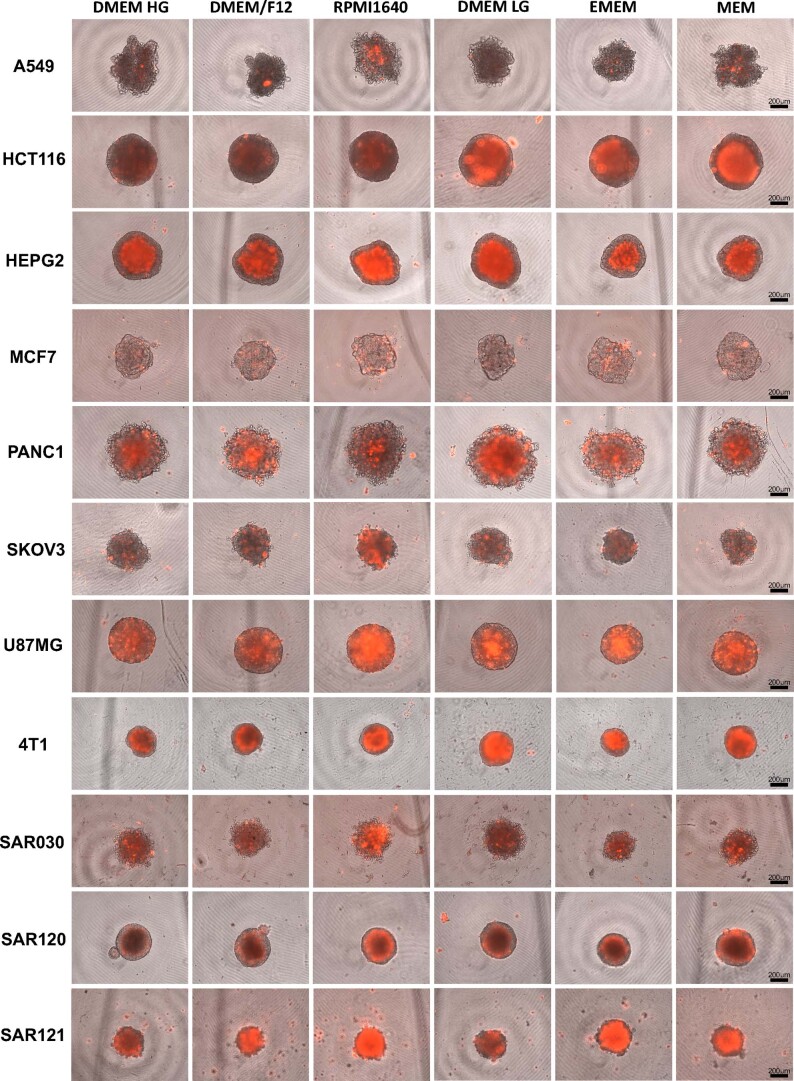
Image presentation of cell death, circularity and size in spheroids cultured in six different media types. Representative microscopy images show ethidium homodimer I stained (red if cell is dead) spheroids of all evaluated cell lines and patient-derived sarcoma cells (cell types indicated on the left) cultured in different media types (indicated on the top) (scale bars 200 µm). Each experiment was repeated independently at least 3 times with 8 technical replicates per experiment, with similar results. Media types are ranked from higher nutrient (left) to lower nutrient (right) richness. Intense staining in the spheroid center is indicative of necrotic core. Next to cell death, images give an indication of circularity and size. Source data

To assess whether the medium-induced transcriptional changes are indicative of distinct cellular properties we examined spheroid metrics including cell death, adenosine triphosphate (ATP) content, ratio of lactate secretion to glucose uptake, secreted protein signatures of angiogenesis and immune interaction, circularity, size, and response to a cancer treatment intervention. These metrics were evaluated in the cell lines covered in the RNA-seq experiments in addition to spheroids derived from tumor types such as liver (HEPG2), human breast (MCF7), pancreas (PANC1), mouse mammary gland (4T1) and sarcoma (early passage patient-derived cultures SAR030, SAR120 and SAR121). Metrics were visualized as a spider plot (as a transformed Z-score), allowing direct comparison of the contribution of each medium for each spheroid type (Fig. 4a). All spheroid metrics were affected by medium type in both established and early passage cell cultures. Medium-induced changes were shared by some of the cell cultures examined while others were cell type specific, underscoring how the heterogeneity of cancer^15,16,31^ can influence the cellular responses to environmental conditions. For example, a consistent increase in cell death was observed in 9 of 11 cell types for RPMI1640, whereas an increase in cell death was observed only in 3 of 11 cell types in the lower nutrient media DMEM LG, EMEM and MEM (Extended Data Fig. 4). In some conditions, cell death occurred particularly in the spheroid center, suggesting necrotic core formation (Extended Data Fig. 5).

**Extended Data Fig. 6:**
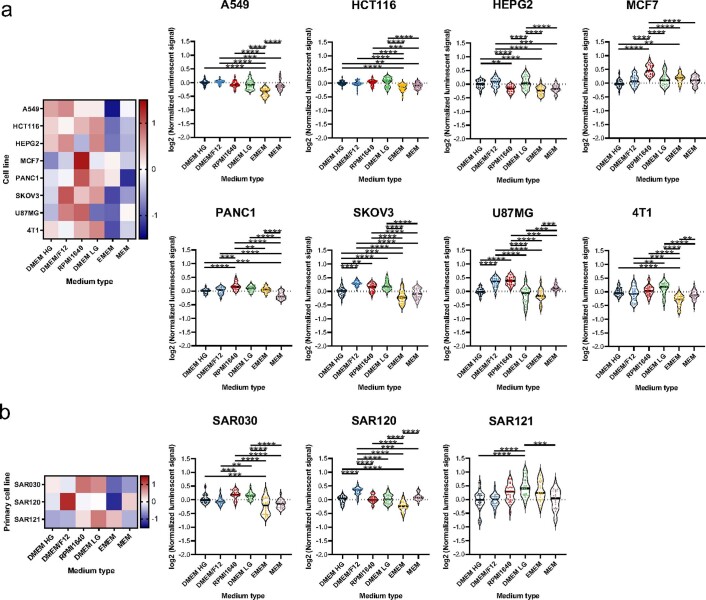
Quantitative presentation of the impact of heterogeneity in culture medium on ATP content in spheroids. Left, Z-score heatmaps and right, violin plots presenting the impact of six different media types on ATP content in (**a**) 8 established cell lines and (**b**) 3 early passage, patient-derived sarcoma cultures. Biological replicates are indicated by a different symbol (N ≥ 3); each symbol is a technical replicate (n = 8). Y-axis represents log2-transformed data, all media types are normalized to DMEM HG. Horizontal bar indicates median. Statistical significance between the groups was determined with a one-way ANOVA and Tukey’s multiple comparison test. **p < 0.01, ***p < 0.001, ****p < 0.0001. Colors in violin plots present media type as in Fig. 3a; media types are ranked from higher nutrient (left) to lower nutrient (right) richness. Source data

**Extended Data Fig. 7:**
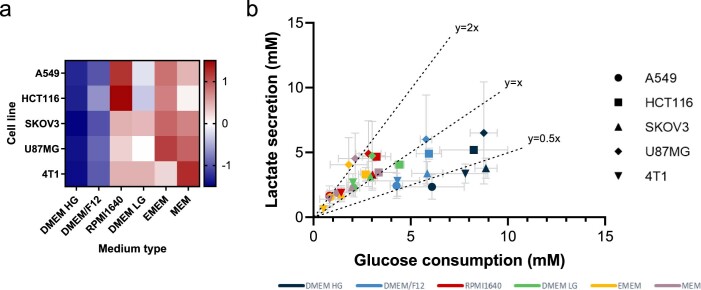
Quantitative presentation of the impact of heterogeneity in culture medium on ratio of glucose uptake to lactate secretion in spheroids. Left, Z-score heatmap and right, scatter diagram presenting the impact of six different media types on ratio of glucose (G) uptake to lactate (L) secretion (L/G ratio) in 5 established cell lines (N ≥ 2, n = 4). Indicative trendlines are presented in the scatter diagram with a dotted line (higher slope means a higher ratio of lactate secretion to glucose uptake (for example y = 2x; for the consumption of one glucose molecule 2 lactate molecules are produced)). Error bars indicate SD. Colors in scatter diagram present media type as in Fig. 3a; media types are ranked from higher nutrient (left) to lower nutrient (right) richness. Source data

Cellular ATP content, as a measure of metabolic activity, was lowest in the nutrient-poor medium EMEM in 8 of 11 cell types (Extended Data Fig. 6). Although commonly related to nutrient deprivation, the cause of necrotic core formation in the multicellular tumor spheroids is still controversial^32^. Nutrient-poor conditions correlated with increased cell death and low ATP content in some cell types (HCT116 and 4T1) but not in all. A549 spheroids cultured in the nutrient-poor media EMEM and MEM had low ATP content but decreased cell death. We measured glucose consumption and lactate secretion in supernatants of spheroids from five cell lines. This L/G ratio was profoundly influenced by medium type in all cell types, with the lowest ratio in the high glucose-containing media DMEM/F12 and DMEM HG (Extended Data Fig. 7). In contrast, two independent studies using 2D cultured cell lines (including A549) did not observe changes in the conversion of glucose to lactate when cultured in different media^13,16^, suggesting the importance of 3D culture-induced nutrient gradients in these metabolic changes.

**Extended Data Fig. 8:**
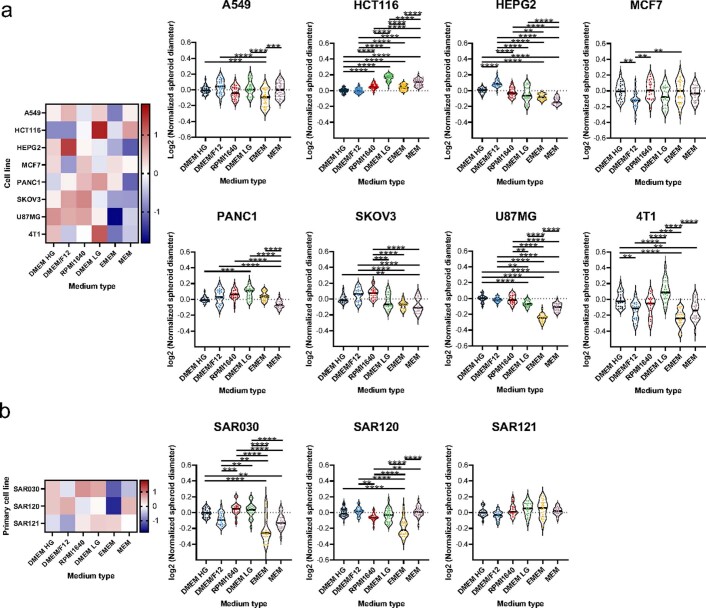
Quantitative presentation of the impact of heterogeneity in culture medium on size of spheroids. Left, Z-score heatmaps and right, violin plots presenting the impact of six different media types on spheroid size difference in (**a**) 8 established cell lines and (**b**) 3 early passage, patient-derived sarcoma cultures. Biological replicates are indicated by a different symbol (N ≥ 3); each symbol is a technical replicate (n = 8). Y-axis represents log2-transformed changes in size, all media types are normalized to DMEM HG. Horizontal bar indicates median. Statistical significance between the groups was determined with a one-way ANOVA and Tukey’s multiple comparison test. **p < 0.01, ***p < 0.001, ****p < 0.0001. Colors in violin plots present media type as in Fig. 3a; media types are ranked from higher nutrient (left) to lower nutrient (right) richness. Source data

Furthermore, culture medium strongly influenced the secretion of proteins implicated in angiogenesis and immune cell interaction (Supplementary Table 5), which are crucial for tumor micro-environment (TME) communication. For example, the angiogenic and immune interactive potential of 4T1 scored high in DMEM LG, but low in DMEM/F12. In contrast, A549 scored a high TME interactivity in DMEM/F12 but a low TME interactivity in DMEM LG (Supplementary Fig. 9). Spheroid morphology, in terms of circularity, ranged between a circularity index of 0.75 and 0.95 in all tested cell lines and differed significantly between cell types (for example, the circularity index of MCF7 was 0.79 ± 0.08 and that of HCT116 was 0.94 ± 0.02). Intriguingly, spheroids cultured in RPMI1640 had the lowest circularity in 7 of 11 cell types (Extended Data Fig. 5 and Supplementary Fig. 10). The metric spheroid size varied significantly with medium type in 10 of 11 cell types analyzed (Extended Data Fig. 8 and Supplementary Table 6). Except for HCT116, MCF7 and SAR121, the nutrient-poor media EMEM or MEM had the smallest spheroids in all cell types analyzed (Extended Data Fig. 5). Size correlated significantly with cellular ATP content (median Pearson’s r = 0.53, two-tailed *P* < 0.0001).

**Extended Data Fig. 9:**
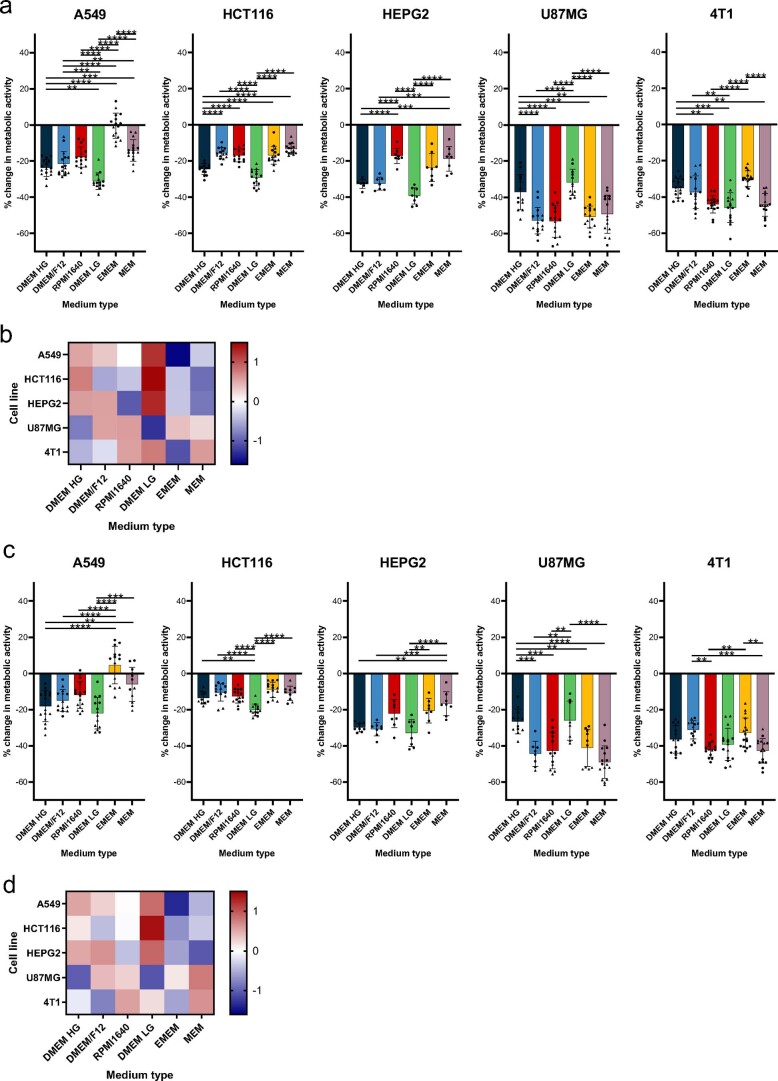
Response to radiotherapy of spheroids cultured in diverse media types. Bar plots (**a** and **c**); and Z-score heatmaps (**b** and **d**) respectively indicate the impact of a single (a and b) 20 Gy and (c and d) 10 Gy fraction on ATP content of spheroids of indicated cell lines cultured in different media types. Biological replicates are indicated by a different symbol (N = 2; except HEPG2 N = 1); each symbol is a technical replicate (n = 8). Statistical significance between the groups was determined with a one-way ANOVA and Tukey’s multiple comparison test. **p < 0.01, ***p < 0.001, ****p < 0.0001. Horizontal bar indicates median, error bars indicate SD. Colors in bar plots present media type as in Fig. 3a; media types are ranked from higher nutrient (left) to lower nutrient (right) richness. Source data

All these medium-induced changes in spheroid metrics warranted investigations into the response to a treatment intervention. Radiotherapy is used in >50% of patients with tumors in sites such as the brain, breast, colorectum, liver and lung^33^, and spheroids are common models to evaluate radiotherapy response^1^. In our evaluated cell lines, the medium type significantly influenced the radiotherapy response. Specifically, 4 of 5 investigated cell lines had the largest response to a 20 Gy fraction in DMEM LG culture medium (Extended Data Fig. 9a,b). The medium-dependent variability in radiotherapy response could not be explained by irreproducibility of the assay. First, biological replicate treatments yielded highly concordant results (median Pearson’s r = 0.90, two-tailed *P* < 0.0001). Second, a 10 Gy fraction had similar patterns of activity across cell lines (Extended Data Fig. 9c,d).

**Extended Data Fig. 10:**
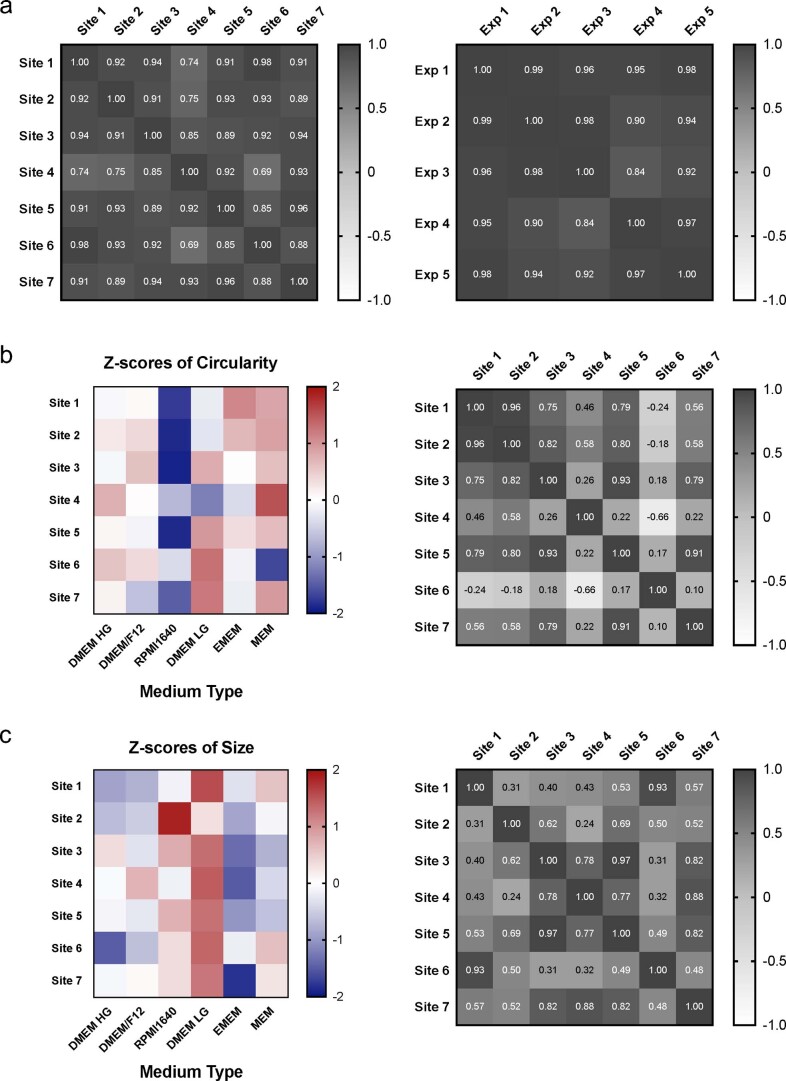
Evaluation of the metrics cell death, circularity and size in an interlaboratory study. (**a**) Correlation matrix of cell death ranking of HCT116 spheroids cultured in six different media types between different participating sites in the interlaboratory study (left) and between biological replicates in the study-initiating laboratory (right). In (**b**) and (**c**), Z-score heatmaps (left) and correlation matrices (right) present the impact of spheroid circularity (**b**) and size (**c**) of HCT116 spheroids cultured in six different media types evaluated across seven different sites in an interlaboratory setting. Media types in Z-score heatmaps are ranked from higher nutrient (left) to lower nutrient (right) richness. The median Spearman correlation across the entire dataset is respectively 0.56 for circularity and 0.57 for size. The larger spread in correlation coefficient of circularity across laboratories is due to subtle absolute differences with most probably limited biological significance. Source data

To validate our results, we initiated an interlaboratory study to chart cell death, circularity and size in HCT116 spheroids cultured in six different media (Fig. 4b). The study used short tandem repeat (STR)-confirmed HCT116 cells, available at each participating laboratory, to allow for a realistic situation in which diversity occurred not only as a result of heterogeneity in culture media but also through differences in passage number, serum batches, pre-spheroid and laboratory-specific culture conditions, researchers and instruments. A dot plot analysis of the ranking of cell death in each analyzed spheroid (Fig. 4b) reproduced a specific pattern of media impact across different sites with a median Spearman correlation across the entire dataset of 0.91. The median correlation between biological replicates at the study-initiating laboratory (site 1) was only slightly higher, at 0.96 (Extended Data Fig. 10a). Furthermore, in agreement with results generated in the study-initiating laboratory, RPMI1640 was validated as the medium in which spheroids had the lowest circularity (in 4 of 6 external sites), and DMEM LG was validated as the medium in which spheroids had the largest size (in 5 of 6 external sites) (Extended Data Fig. 10b,c). Consequently, the interlaboratory data on HCT116 spheroids were indicative of the robustness of the impact of medium heterogeneity on spheroid metrics and demonstrated the generalizability of our findings.

**Fig. 5:**
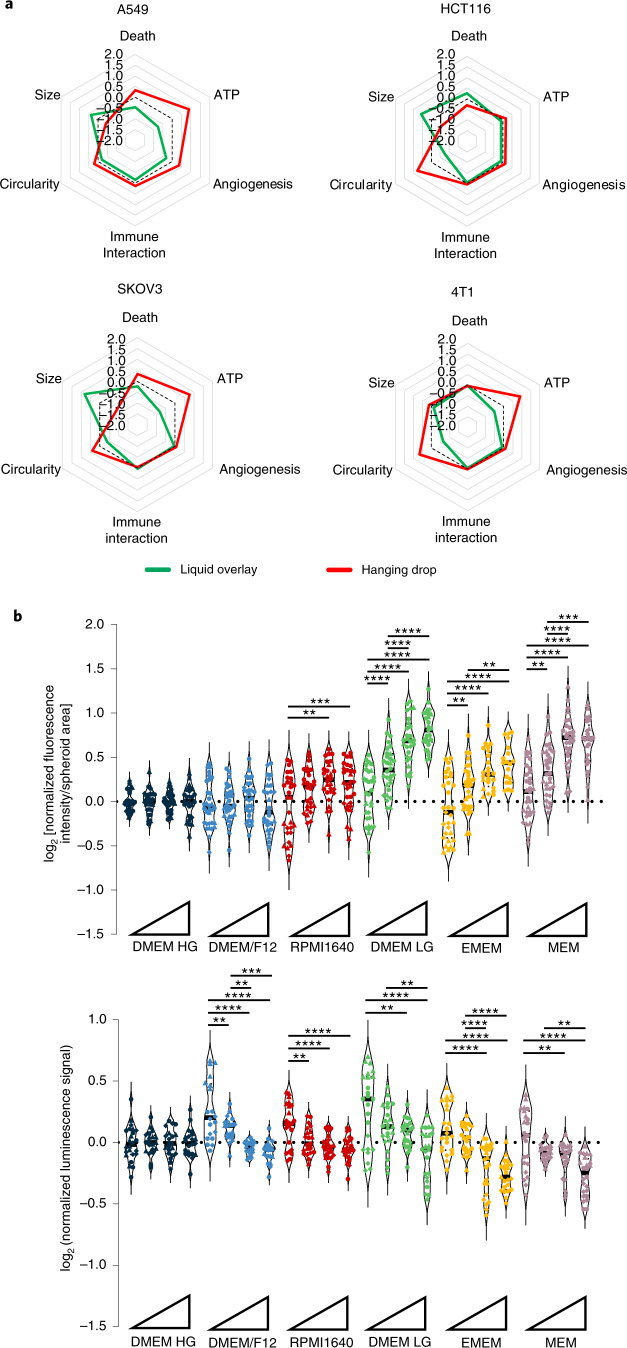
Effect of formation methodology and spheroid size-induced heterogeneity on spheroid metrics across multiple cell types. **a**, Spider plot of metrics from spheroids of indicated cell lines generated by the hanging drop (red) or liquid overlay (green) spheroid formation method. Axes represent the Z-score metrics of cell death, ATP content, secreted protein signatures of angiogenesis and immune interaction, circularity and size. A higher Z-score means a higher metric value. **b**, Violin plots representing the impact of spheroid size on cell death (upper panel) and ATP content (lower panel) metrics of HCT116 spheroids cultured in six different media. Each biological replicate has a different symbol (*N* ≥ 3), and each symbol is a technical replicate (*n* = 8). Triangles at the X axis represent increasing seeding cell number and consequently increasing spheroid size; for absolute size estimates see Supplementary Table 6. The Y axis represents log_2_-transformed data, and all media are normalized to DMEM HG. The horizontal bar indicates the median. Statistical significance between the groups was determined with a one-way ANOVA and Tukey’s multiple comparison test. **P < 0.01, ***P < 0.001, ****P < 0.0001. Colors indicate medium type; media are ranked from higher nutrient (left) to lower nutrient (right) richness. Source data

An impact of spheroid formation methods on morphology-related spheroid metrics has been shown clearly in previous reports^28–30,34^. The two most frequently reported formation methods (hanging drop and liquid overlay using ULA plates) in A549, HCT116, SKOV3 and 4T1 cell cultures (Supplementary Figs. 5 and 8) were compared in one medium type (DMEM LG), and this showed that ATP content, circularity and size were profoundly affected by formation methodology in a cell type-specific manner (Fig. 5a and Supplementary Fig. 11).

Spheroid size, a parameter influencing hypoxia and necrotic core formation^1^, is largely underreported in MISpheroID. Spheroids with different seeding cell numbers at the start of the experiment had size-dependent changes in the metrics cell death and ATP content (Fig. 5b, Supplementary Figs. 12 and 13 and Supplementary Table 6), which confirmed previous reports of the importance of spheroid size on spheroid characteristics^10,11^. Differences were observed in most culture media for all cell types evaluated. Intriguingly, the smallest (<550 µm) HCT116 spheroids showed medium-dependent changes in ATP but not in cell death. Thus, depending on cell line, spheroid size differentially affected spheroid metrics within a certain medium type but also between medium types.

The spheroid community is challenged by heterogeneity and reporting deficiencies that cause a significant variation in the readout of one or more spheroid metrics in a cell type-specific manner. Our analyses underscore the importance of transparent reporting of experimental parameters affecting spheroid interpretation.

### Creation of the MISpheroID tool

Transparency expectations to ensure experimental robustness and reproducibility have prompted the creation of reporting tools in various fields^17,18,21–26^. In accordance with MISpheroID knowledgebase interrogation, in-depth empirical evaluation and available supportive literature^10–13,28–30,34^, the MISpheroID Consortium recommends the introduction of the MISpheroID tool in the spheroid community. This tool generates a spheroid ID string consisting of four components that the Consortium argues to be the minimum information required for interpretation, comparison and replication of spheroid experiments: cell type(s) (one or multiple cell types (co-culture) included in one spheroid), culture medium (the environment in which spheroids are formed and cultivated), spheroid formation method (that is, liquid overlay (for example, ULA plates), hanging drop, spinner flask, microfluids and so on), and size (the diameter ± s.d. of the spheroid after spheroid formation (at the moment of application)).

#### Typical example of an MISpheroID string

A representative example of an MISpheroID string is [4T1 – EMEM – Liquid overlay – 348 ± 23 µm].

Currently, only 300 of 1,628 (18.4%) breast cancer-related spheroid experiments provide all four components of the MISpheroID string. Global reporting of MISpheroID string parameters shows that some are reported relatively more often (that is, cell line and spheroid formation method) than others (that is, culture medium and size). Importantly, in 80.7% of the experiments, an increase in MISpheroID string reporting can be achieved without additional experiments (correct reporting of medium type and size, based on available microscopy images).

### Using the MISpheroID platform

**Fig. 6:**
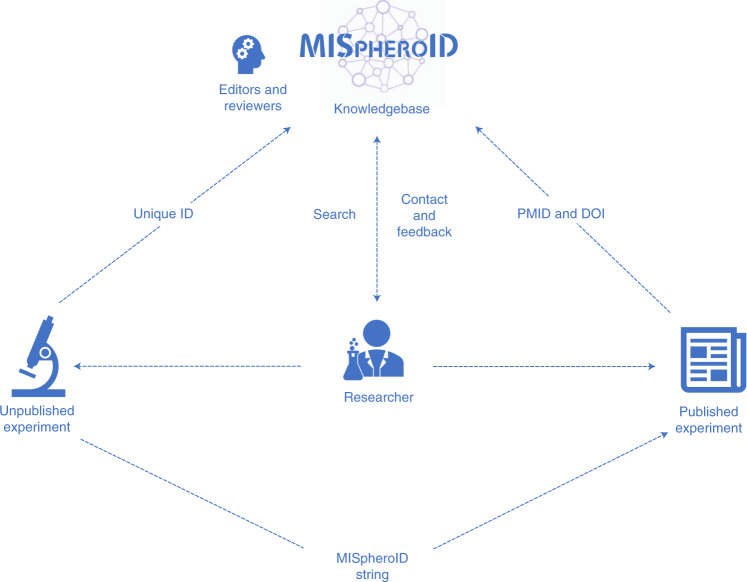
Implementation of the MISpheroID knowledgebase. This flowchart illustrates the application of MISpheroID.

We invite the community to upload spheroid experiments through https://www.mispheroid.org (Fig. 6). As part of each upload, 31–55 (depending on sub-questions) experimental parameters related to cell type, culture medium, spheroid formation method and size are recorded and are fully compatible with queries for deeper information. Each annotated experiment receives a MISpheroID string and unique registration code. The MISpheroID string does not reflect the quality of a spheroid experiment, nor does it impose a specific methodology, but it improves spheroid research transparency and experimental design. Users can search the knowledgebase for articles using a range of search parameters. The query result list is accompanied by a MISpheroID string and Pubmed ID (PMID). MISpheroID querying stimulates awareness and motivates researchers to compare general and specific information. We encourage researchers to provide feedback about the knowledgebase and transparency tool using the contact section of MISpheroID (Fig. 6).

## Discussion

Spheroids are attractive 3D tissue structures for research purposes, and rapid progress in imaging, automated high-throughput production and microfluidic technologies ensures their future implementation in drug screening, tumor biology studies and tissue engineering. However, to reach its full potential, there must be in-depth reporting of the diverse experimental parameters in the methodological setup of spheroids. This study objectively demonstrates that cell line, culture medium composition, spheroid formation method and spheroid size significantly affect the phenotypic landscape of spheroids. Although it was not the Consortium’s aim to identify the causes of the culture medium-induced changes in spheroid metrics, we expect that the non-physiological concentrations of some nutrients affect 3D biology in a cell context-dependent manner. The choice of medium type should be considered when designing studies that aim to explore pathophysiology or that aim to identify the dependency of cancer cells on specific signaling pathways, or when investigating the effects of therapeutic intervention (such as radiotherapy in this study). Most importantly, failure to understand heterogeneity can result in data that are difficult to interpret and reproduce.

The MISpheroID tool reflects the process of generating a spheroid to allow evaluation of experimental consistency and straightforward resource exchange for understanding spheroid data output (biology, drug screening and so on). Some of the challenges (cell line and culture medium) are not unique to the spheroid field and have been discussed previously^21–23^. We recognize that achieving a meaningful improvement in the transparency of reporting will require engagement and acceptance from all stakeholders, including investigators, reviewers, funding agencies and journal editors^17,18,25^.

The MISpheroID resource has its limitations, which should be considered. First, empirical data are obtained from monoculture spheroids; co-cultures were not analyzed in this study. Second, spheroid metrics were evaluated at a fixed predefined time point; longitudinal analyses were not included in the study. Third, the knowledgebase covers a high number of spheroid experiments representing cancer biology but does not cover spheroid experiments from other fields, thereby potentially underestimating the full spheroid landscape. Despite these limitations, the knowledgebase contains information on non-cancer cells present in spheroid co-culture experiments such as mesenchymal and immune cells. Considering differences in metabolic demands for non-cancer cells, the components of the MISpheroID knowledgebase and tool are equally useful for non-cancer cells. Other emerging 3D model systems, such as organoids and self-renewing multicellular aggregates that self-organize into lumen-containing ex-vivo organs, may also benefit from the MISpheroID tool to improve transparency and document heterogeneity^35^.

We developed MISpheroID to capture and disseminate data related to spheroid models and we will continue to evolve MISpheroID to reflect the state of the art in the field. Given the recent success of immune checkpoint inhibitors in the treatment of cancer, improving the immune component of spheroid models can be steered with knowledge accumulated in this study such as protein secretion in spheroid supernatants (for specific secretion profiles see the source data files). Recent advancements in technology will facilitate more in-depth characterization and reporting of spheroid metrics. For example, breakthroughs in submillimeter particle characterization in geology, engineering and the food industry may lead to better 3D characterization of spheroid sphericity and compaction^36^.

As has been demonstrated across multiple domains, adoption of minimum information by a research community accelerates the rate of transparency and drives scientific progress. Reporting of the minimum information required is already compulsory or recommended for publishing in several journals^24^. MISpheroID contributes to the field of 3D spheroid biology by providing first, a knowledgebase that catalogs spheroid setup, characterization and applications to enable the community to share and access key technical and biological insights in 3D experimentation; second, minimum information parameters combined with a tool to implement them; and last, a resource containing experimental data demonstrating the impact of experimental variations on spheroid metrics. In conclusion, the MISpheroID Consortium aims to advance 3D biology in both academic and industrial environments by removing the barriers of inconsistency while promoting reproducibility.

## Methods

### Literature search

Medline (PubMed) was searched for articles applying active spheroid formation methods with cancer cells (cell lines and early passage patient-derived cell cultures). Therefore, the following key terms were applied: spheroid (and derived terms), cancer, neoplasm, tumor and ‘organ’ (brain, breast, colorectum, liver, lung, ovary and pancreas). We focused on active spheroid formation, therefore at abstract check, reviews and letters were excluded as well as articles that did not involve active spheroid formation or those in which spheroids did not include organ-specific cells. At full-text screening, we applied the same exclusion criteria and excluded articles for which the full text was not available. Spheroids from multiple cell lines or spheroid formation methods were considered as a separate experiment. For the breast cancer spheroid-related articles used to set up the knowledgebase, published articles from 1979 to 2020 were evaluated. For the spheroid-related articles from other organ tumor types, published articles from 2018 to 2021 were evaluated. As an example, a detailed flow chart for literature screening of breast of breast cancer spheroids is provided in Supplementary Fig. 1.

### Experimental setup

An overview schematic (Supplementary Fig. 14) explains the use of different cell types, methodological setups and metrics evaluated in the study as detailed in the Methods section.

### Cell culture

The cell lines A549 (cat. no. CCL-185), HCT116 (cat no. CCL-247), HEPG2 (cat. no. HB-8065), MCF7 (cat. no. HTB-22), PANC1 (cat. no. CRL-1469), SKOV3 (cat. no. HTB-77), U87MG (cat. no. HTB-14) and 4T1 (cat. no. CRL-2539) were purchased from the American Type Culture Collection. To establish the early passage cell cultures (SAR030, SAR120 and SAR121), patient-derived sarcoma samples (Supplementary Table 7) were cut into pieces measuring 1–2 mm^3^. The pieces were digested in 500 U ml^−1^ collagenase II solution (cat. no. 17101015, ThermoFisher) and 22 KU ml^−1^ DNase I solution (cat. no. A3778.0010, VWR) and processed according to the Gentlemax tumor digestion protocol (Miltenyi Biotec). The cell suspension was applied to a cell strainer (100 μm, cat. no. 352360, Corning), centrifuged at 300×g for 5 min and, after aspiration of the supernatant and removal of remnant red blood cells, maintained in culture. All human cell lines were authenticated using a 21-Marker STR Profile test (Eurofins) and tested monthly using the Mycoalert Mycoplasma Detection Kit (cat. no. LT07-318, Lonza) to exclude mycoplasma contamination. All cells were cultured in DMEM (cat. no. 41965039, ThermoFisher) supplemented with 10% heat-inactivated fetal bovine serum (FBS) (cat. no. ATCC-30-2030, LGC Standards), 100 IU ml^−1^ penicillin and 100 mg ml^−1^ streptomycin (cat. no. 15070063, ThermoFisher). Cells were expanded and maintained as a monolayer at 37 °C in an atmosphere of 5% CO_2_ in air and passaged at 80% confluence.

### Spheroid formation

The U-shaped, 384-well ULA plates (cat. no. MS-9384UZ, S-bio) were seeded with a suspension of 80 µl cell culture media with 2 × 10³ cells per well (A549, HCT116, HEPG2, MCF7, PANC1, SKOV3, U87MG, 4T1, SAR120, SAR121) or 8 × 10³ cells per well (SAR030) in 2 × 8 technical replicates per condition. The culture media used were DMEM HG (4.5 g l^−1^ = 25 mM glucose) (cat. no. 41965039, ThermoFisher), DMEM/F12 (1:1) (3.15 g l^−1^ = 17.5 mM glucose) (cat. nos. 41965039 and 21765029, ThermoFisher), RPMI1640 (2 g l^−1^ = 11.1 mM glucose) (cat. no. 21875091, ThermoFisher), DMEM LG (1 g l^−1^ = 5.6 mM glucose) (cat. no. 31885023, ThermoFisher), EMEM (1 g l^−1^ = 5.6 mM glucose) (cat. no. 10-009-CV, Corning) and MEM (1 g l^−1^ = 5.6 mM glucose) (cat. no. 10370-047, ThermoFisher), all supplemented with 10% FBS, 100 IU ml^−1^ penicillin and 100 mg ml^−1^ streptomycin. Note that 5.6 mM glucose is the physiological plasma glucose concentration. MEM was supplemented with 2 mM l-glutamine. Extended Data Fig. 3 provides extensive details on the nutrient content of 10 culture media reported in MISpheroID, of which six were empirically evaluated. The 384-well ULA plates were sealed with Breathe-Easy semipermeable tape (cat. no. BEM-1, Diversified Biotech) to prevent evaporation. The spheroids were cultured at 37 °C in an atmosphere of 5% CO_2_ under normoxia.

For the comparison of spheroid size, 2 × 10^3^, 4 × 10^3^, 6 × 10^3^ or 8 × 10^3^ cells (A549 and SKOV3) and 0.5 × 10^3^, 1 × 10^3^, 2 × 10^3^ or 3 × 10^3^ cells (HCT116) were seeded in 80 µl cell culture media in the U-shaped, 384-well ULA plates. The culture media used were DMEM HG, DMEM/F12, RPMI1640, DMEM LG, EMEM and MEM.

For the comparison of the liquid overlay method and the hanging drop method, 2 × 10^3^ cells (A549, HCT116, SKOV3, 4T1) were seeded in 80 µl cell DMEM LG in U-shaped 96-well ULA plates (cat. no. CLS7007-24EA, Sigma-Aldrich) in the liquid overlay method. In the hanging drop method, 2 × 10^3^ cells were plated under the lids of petri dishes (cat. no. A19618, Novolab) in 20 µl drops of DMEM LG (50 technical replicates). The bottom of the petri dishes was filled with 10 ml PBS (phosphate buffered saline; cat. no. 20012019, ThermoFisher) to limit evaporation.

### RNA sequencing

Spheroids from four independent cell lines using four technical replicates from one biological replicate were used and cultured in 384-well ULA plates in six different cell culture media (a total of 96 conditions) for 5 d (HCT116) or 7 d (A549, SKOV3 and U87MG). RNA extraction was performed on two spheroids per condition using the miRNeasy Micro Kit (cat. no. 217084, Qiagen). RNA-seq libraries were prepared from purified RNA using the QuantSeq 3’ mRNA-Seq Library Prep Kit FWD for Illumina (Lexogen) according to the manufacturer’s instructions, with 27.5 ng RNA that was treated with Heat-Labile Double Strand-specific DNase (Arcticzymes). The individual libraries were quantified by qPCR using the KAPA Library Quantification Kit (Roche) and equimolarly pooled. The pool concentration was measured with Qubit, and sequencing was carried out at a concentration of 1.4 pM with 1% PhiX on a NextSeq 500 (Illumina) (NextSeq software v.4.0.1) using a high-output 1 × 75 run. Reads were mapped to the human genome using Tophat and gene expression counts were generated using HTSeq.

### Data normalization and gene set enrichment analysis

Normalization and differential gene expression analysis were performed using DESeq2 (v1.30.1). Terms from the Molecular Signatures Database (MSigDB) Hallmark Gene Signatures^27^ were used for enrichment analysis with GSEA software (v4.1.0). We tested gene sets for significant enrichment among the differentially expressed genes between the six medium types used to culture the spheroids in each cell line, to assess how they might differ from each other in terms of molecular pathways. Statistical significance between the different media for all hallmarks was determined for every cell line using one-way analysis of variance (ANOVA) and Tukey’s multiple comparison test with the alpha level of significance set at 0.05. Principal component analysis with the Manhattan distance index was performed using PAST4.03.

### Cell death staining

Spheroids were cultured in the 384-well ULA plates in the different cell culture media for 5 d (HCT116) or 7 d (all other cell lines). For the comparison of the liquid overlay method and the hanging drop method, spheroids were cultured for 3 d. A total of 60 µl medium per well was replaced with 10 µM Ethidium homodimer I solution (cat. no. 300519, Santa Cruz) in PBS supplemented with Ca^2+^ and Mg^2+^. The hanging drop spheroids were transferred to the 96-well ULA plate and 60 µl 10 µM Ethidium homodimer I solution was added. The fluorescence signal was observed after 15 min incubation time at 20 °C room temperature with an Axiovert 200 M fluorescence microscope (Carl Zeiss) using AxioVision release 4.8. The average dead signal was measured over the spheroid area in ImageJ (v1.52v.). Analysis was performed on a minimum of three biological replicates, with each biological replicate having eight technical replicates per condition. Statistical significance between the groups was determined with a one-way ANOVA and Tukey’s multiple comparison test with the alpha level of significance set at 0.05.

### Interlaboratory study

The study-initiating laboratory sent culture media, 384-well ULA plates and Breathe-Easy semipermeable tape to six different laboratories that were part of the international MISpheroID Consortium. To perform the experiments, the laboratories added their own batches of serum to the culture media. All cells were authenticated by STR profiling. To conduct the dead staining, laboratories applied in-house optimized dead staining protocols, after which the raw data concerning dead signal, spheroid circularity and size were analyzed by the study-initiating laboratory.

### ATP assay with CellTiter-Glo 3D

Spheroids were cultured in 384-well ULA plates in the different cell culture media for 5 d (HCT116) or 7 d (all other cell lines). For the comparison of the liquid overlay method and the hanging drop method, spheroids were cultured for 3 d. Individual spheroids in the culture medium were pipetted into white micro 96-well plates (cat. no. 236108, ThermoFisher) and an equal volume of CellTiter-Glo 3D (cat. no. G9683, Promega) reagent was added. The contents were mixed for 5 min on an orbital shaker to induce cell lysis, while shielded from light. Luminescence readout (Gen5 Data Analysis Software v3.08.01) was performed after 25 min incubation at 20 °C (room temperature). Analysis was performed on a minimum of three biological replicates, with each biological replicate having eight technical replicates per condition. Statistical significance between the groups was determined with a one-way ANOVA and Tukey’s multiple comparison test with significance level alpha 0.05.

### Irradiation

Spheroids were cultured in the 384-well ULA plates in the different cell culture media for 3 d before being irradiated with 10 or 20 Gy single fractions with 6 MV photons from an Elekta Synergy linear accelerator (Elekta). Irradiation response was measured and calculated after 2 d (HCT116) or 4 d (SKOV3, U87MG and 4T1) with the percentage change in metabolic activity (CellTiter-Glo 3D) compared with non-irradiated control spheroids. Analysis was performed on a minimum of eight technical replicates per condition. Statistical significance between the groups was determined with a one-way ANOVA and Tukey’s multiple comparison test with the alpha level of significance set at 0.05.

### Measurement of glucose and lactate

Endpoint concentrations of glucose and lactate in the spheroid supernatants were measured using enzymatic assays involving bioluminescent NADH detection technology and a selective dehydrogenase (Glucose-Glo J6021 and Lactate-Glo J5021 assay; Promega). For the glucose-Glo assay, supernatants of spheroids cultured in DMEM HG and DMEM/F12 were diluted 1:500 in PBS, and supernatants of spheroids cultured in RPMI1640, DMEM LG, EMEM and MEM were diluted 1:200 in PBS. For the lactate-Glo assay, supernatants of spheroids were diluted 1:100 in PBS. Controls (medium without cells) were diluted correspondingly. A 50 µl sample was pipetted into white micro 96-well plates (cat. no. 236108, ThermoFisher) and an equal volume of assay reagent was added. The contents were mixed for 30 s on an orbital shaker while shielded from light. Luminescence readout (Gen5 Data Analysis Software v3.08.01) was performed after 1 h incubation at room temperature. Glucose consumption and lactate production were calculated by subtraction of the glucose or lactate concentrations of the medium without cells. Analysis was performed on a minimum of two biological replicates with each biological replicate having four technical replicates per condition. Statistical significance between the groups was determined with a Kruskal–Wallis test with Dunn’s correction, with the alpha level of significance set at 0.05.

### Luminex

Spheroids were cultured in the 384-well ULA plates in the different cell culture media for 5 d (HCT116) or 7 d (all other cell lines). For the comparison of the liquid overlay method and the hanging drop method, spheroids were cultured for 3 d. Eight technical replicates (liquid overlay method) or 42 technical replicates (hanging drop method) were then collected and the supernatant was passed through a 0.2 µm filter (cat. no. A37111, Novolab) and processed with the Human Cytokine/Chemokine Array 71-Plex Panel and the Human Angiogenesis Array and Growth Factor 17-Plex Array by Eve Technologies. The supernatant collected from the 4T1 mouse mammary gland cell line was processed with the Mouse Cytokine/Chemokine Array 44-Plex Panel. Analysis was performed on a minimum of three biological replicates per condition with subtraction of the growth factor concentrations of medium without cells. Growth factors were grouped based on their GO-Biological processes related to immune interaction or angiogenesis via the UniProt Knowledgebase.

### Size and circularity calculations

Spheroid size (diameter) and circularity were determined using AnaSP v1.4 (ref. ^37^). The spheroid size was described by the equivalent diameter (ED). The ED is the diameter of a circle with the same projected area as the spheroid, calculated by

1 $${{{\mathrm{ED}}}} = 2\surd \left( {{{{\mathrm{area}}}}/\pi } \right)$$

The spheroid circularity was determined by the formula:

2 $${{{\mathrm{circularity}}}} = 4\pi \times{{{\mathrm{area}}}}/\left( {{{{\mathrm{convex}}}}\,{{{\mathrm{perimeter}}}}} \right)^2$$

### Z-score calculations

Z-scores were calculated in Excel (v.2106) according to the formula

$${{{\mathrm{Z}}}} = \left( {{{x}} - \mu } \right)/\sigma$$

with *x* being the value of every replicate, *µ* being the average of the population (all replicates over the different media), and *σ* being the standard deviation of the population (all replicates over the different media).

The Z-score of a culture medium was subsequently calculated as the average of the Z-scores for that culture medium.

### Outlier exclusion

Data points were first excluded manually (for example, spheroids containing a dust particle when measuring dead signal, spheroids that were lost when pipetting to a white plate for measuring metabolic activity with CellTiter-Glo 3D and so on). Afterwards, outliers were calculated via the ROUT method (Q = 1%) with Graphpad Prism v8.4.3, taking into account that the number of datapoints plotted in the graphs can be lower than *N* × *n* due to outlier exclusion.

### Statistical analysis

Statistical analysis was performed in Graphpad Prism v8.4.3. Unpaired comparisons were conducted using a one-way ANOVA or Kruskal–Wallis test (after the mentioned transformations and the Shapiro–Wilk assessment for normality) with Tukey’s or Dunn’s multiple comparison test. Comparisons of the spheroid formation method were conducted using unpaired Student’s *t*-test with Welch’s correction. Correlation analysis was conducted by determination of Pearson or non-parametric Spearman correlation coefficients. The significance level for all tests was 0.05, however, in the Figures the statistical significance is shown only for significance levels ≤ 0.01.

### Reporting Summary

Further information on research design is available in the Nature Research Reporting Summary linked to this article.

## Online content

Any methods, additional references, Nature Research reporting summaries, source data, extended data, supplementary information, acknowledgements, peer review information; details of author contributions and competing interests; and statements of data and code availability are available at 10.1038/s41592-021-01291-4.

## Supplementary information

Supplementary Information Supplementary Figs. 1–14, Supplementary Tables 1–7.

Reporting Summary

Supplementary Table 2 Checklist of 98 reporting parameters concerning experiment identification, spheroid set up, spheroid characterization and application.

Supplementary Data 1 Luminex data from cytokines clustered as angiogenesis and immune interaction.

Supplementary Data 2 Circularity data.

Supplementary Data 3 Data showing impact of formation method on spheroid metrics.

Supplementary Data 4 Data showing impact of size and culture medium on spheroid metrics.

Supplementary Data 5 Data images showing impact of size and culture medium.

Supplementary Data 6 Sizes of spheroids.

Supplementary Data 7 Statistical source data of all Figures, Extended Data Figures and Supplementary Figures.

## Source data

Source Data Fig. 1 Systematic literature, applied for Fig. 1, Extended Data Fig. 1 and Supplementary Figs. 1–6.

Source Data Fig. 2 Systematic literature data applied for Fig. 2 and Extended Data Fig. 2.

Source Data Fig. 3 RNA-seq data.

Source Data Fig. 4 For the raw data of Fig. 4a please see the raw data files of Extended Data Figs. 4 (cell death), 6 (ATP), 7 (L/G), 8 (size) and 9 (therapy response) and Supplementary Figs. 9 (angiogenesis and immune interaction) and 10 (circularity). For the raw data of Fig. 4b please see the raw data file of Extended Data Fig. 10.

Source Data Fig. 5 Liquid overlay versus hanging drop.

Source Data Extended Data Fig. 1 Systematic Literature, applied for Fig. 1, Extended Data Fig. 1 and Supplementary Figs. 1–6.

Source Data Extended Data Fig. 2 Systematic literature data applied for Fig. 2 and Extended Data Fig. 2.

Source Data Extended Data Fig. 4 Cell death analysis.

Source Data Extended Data Fig. 5 Cell death images.

Source Data Extended Data Fig. 6 ATP analysis.

Source Data Extended Data Fig. 7 Metabolism glucose and lactate analyses.

Source Data Extended Data Fig. 8 Spheroid size.

Source Data Extended Data Fig. 9 Therapy response.

Source Data Extended Data Fig. 10 Multisite study

## Data Availability

RNA-seq data generated during the current study are available in the ArrayExpress database (www.ebi.ac.uk/arrayexpress) under accession number E-MTAB-10862. The source data underlying Figs. 1–5, Extended Data Figs. 4–10, Supplementary Figs. 9–13 and Supplementary Table 6 are provided as a source data file. All literature study data are available through the MISpheroID knowledgebase. Source data are provided with this paper.

## Extended data

are available for this paper at 10.1038/s41592-021-01291-4.
