# Supplementary material for: MISpheroID: a knowledgebase and transparency tool for minimum information in spheroid identity
Source: Nat Methods. 2021 Nov 1;18(11):1294–303. doi: 10.1038/s41592-021-01291-4 (PMC8566242; doi:10.1038/s41592-021-01291-4)
Supplement: Supplementary file 1 — Supplementary Figs. 1–14, Supplementary Tables 1–7. [file 41592_2021_1291_MOESM1_ESM.pdf]

nature portfolio

https://doi.org/10.1038/s41592-021-01291-4

Supplementary information

MISpheroID: a knowledgebase and transparency tool for minimum information in spheroid identity

In the format provided by the authors and unedited

Supplementary Figures

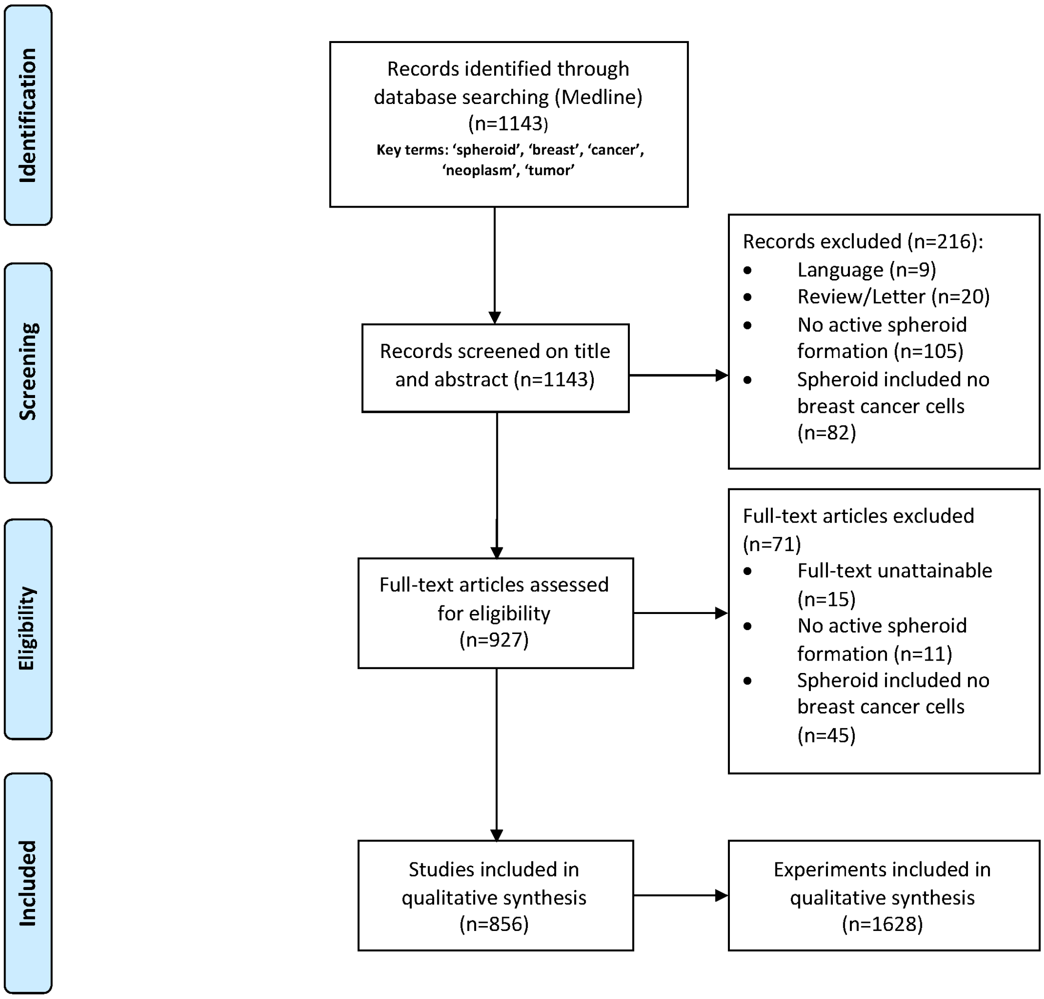

Supplementary Figure 1. Creation of the MISpheroID knowledgebase with focus on breast cancer-derived spheroids

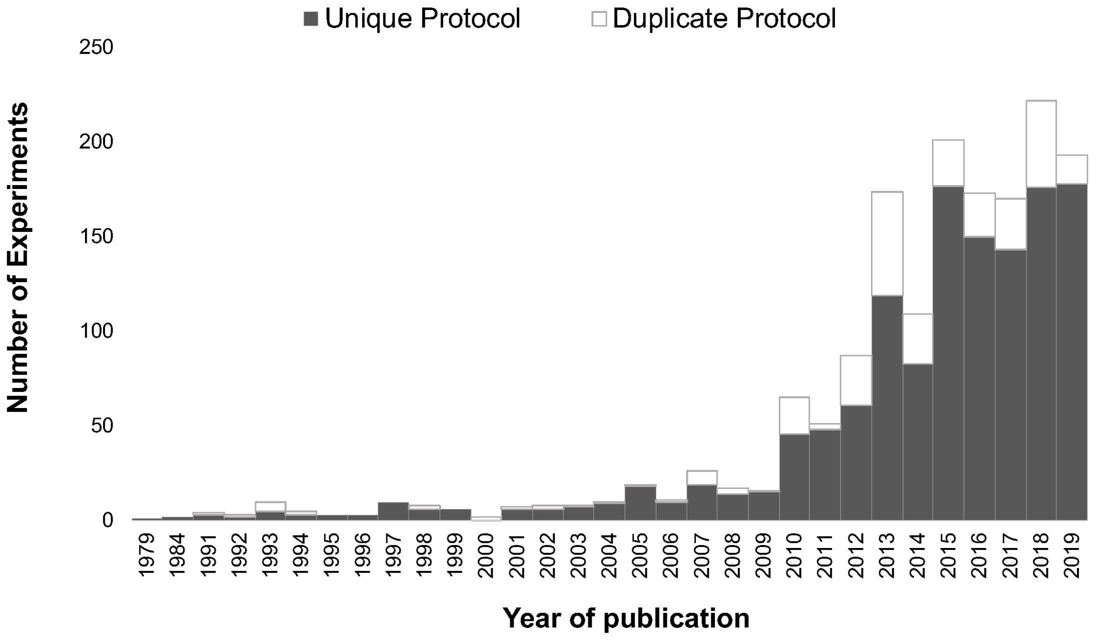

Supplementary Figure 2. Heterogeneity of breast cancer spheroid experiments

Stacked bar chart showing the total number of breast spheroid experiments over time indicating the contribution of unique protocols versus duplicate protocols. Hence, most new spheroid experiments implement a unique (and thus new) protocol setup. Experimental settings that are considered in this analysis are: cell line(s), culture medium, serum concentration, glucose concentration, culture medium supplements, spheroid formation method, scaffold type, liquid overlay type, culture plate coating (e.g. agarose), plate size (i.e. number of wells), well bottom shape and centrifugation step.

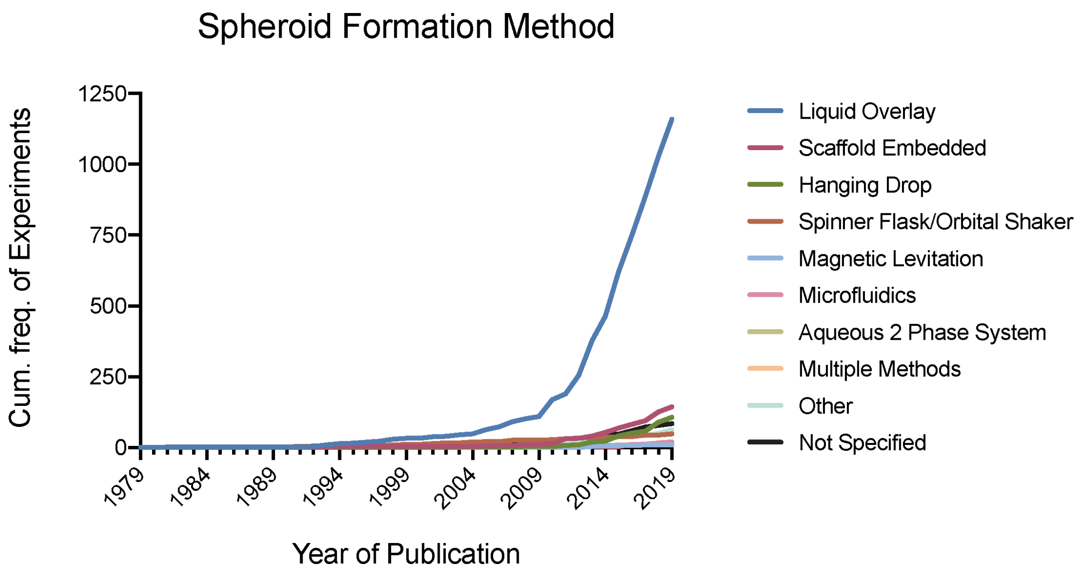

Supplementary Figure 3. Evolution of spheroid formation methodology over time

Cumulative frequency plot showing the cumulative number of experiments using a specific spheroid formation method to create breast cancer spheroids.

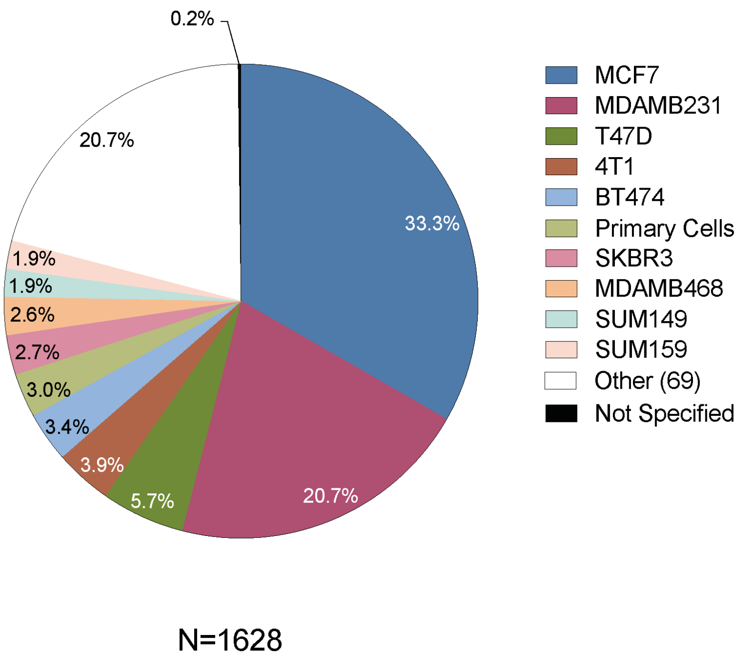

Supplementary Figure 4. Breast cancer cell line distribution in spheroid experiments

Pie chart showing the proportion of cell lines used to prepare breast cancer spheroids.

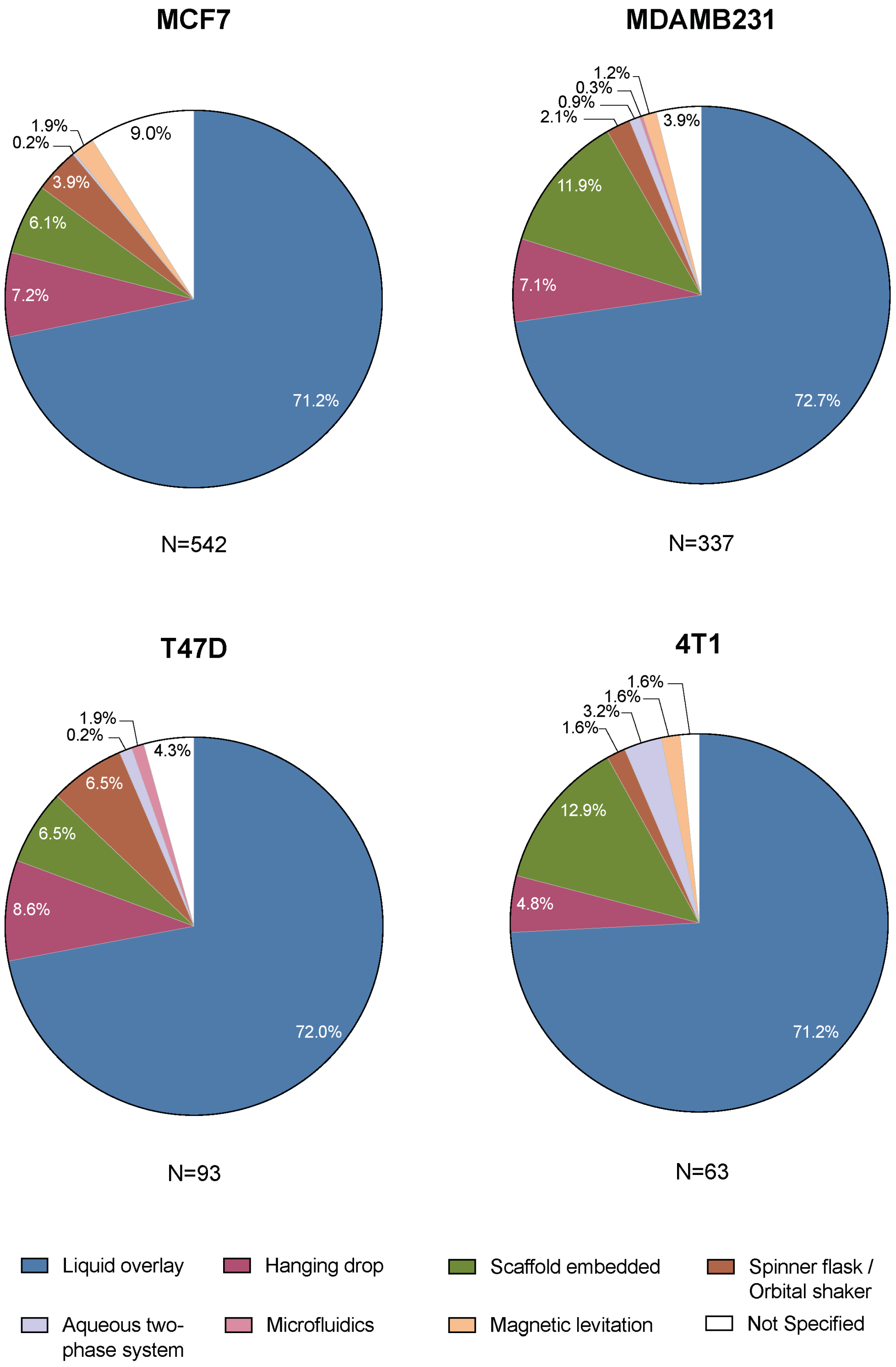

Supplementary Figure 5. Distribution of the applied spheroid formation methodology to prepare spheroids of the estrogen dependent MCF7 and T47D, and triple-negative MDAMB231 and 4T1 breast/mammary gland cancer cell lines

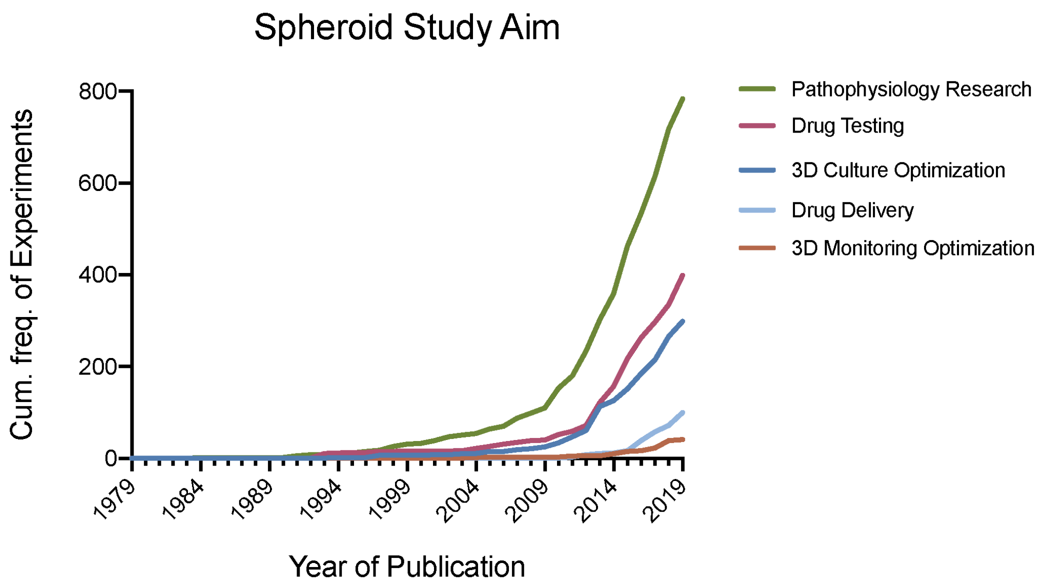

Supplementary Figure 6. Study aim of spheroid experiments

Cumulative frequency plot showing the evolution of the cumulative number of study aims of breast cancer spheroid research publications from 1979 – 2020 as recorded by MISpheroID.

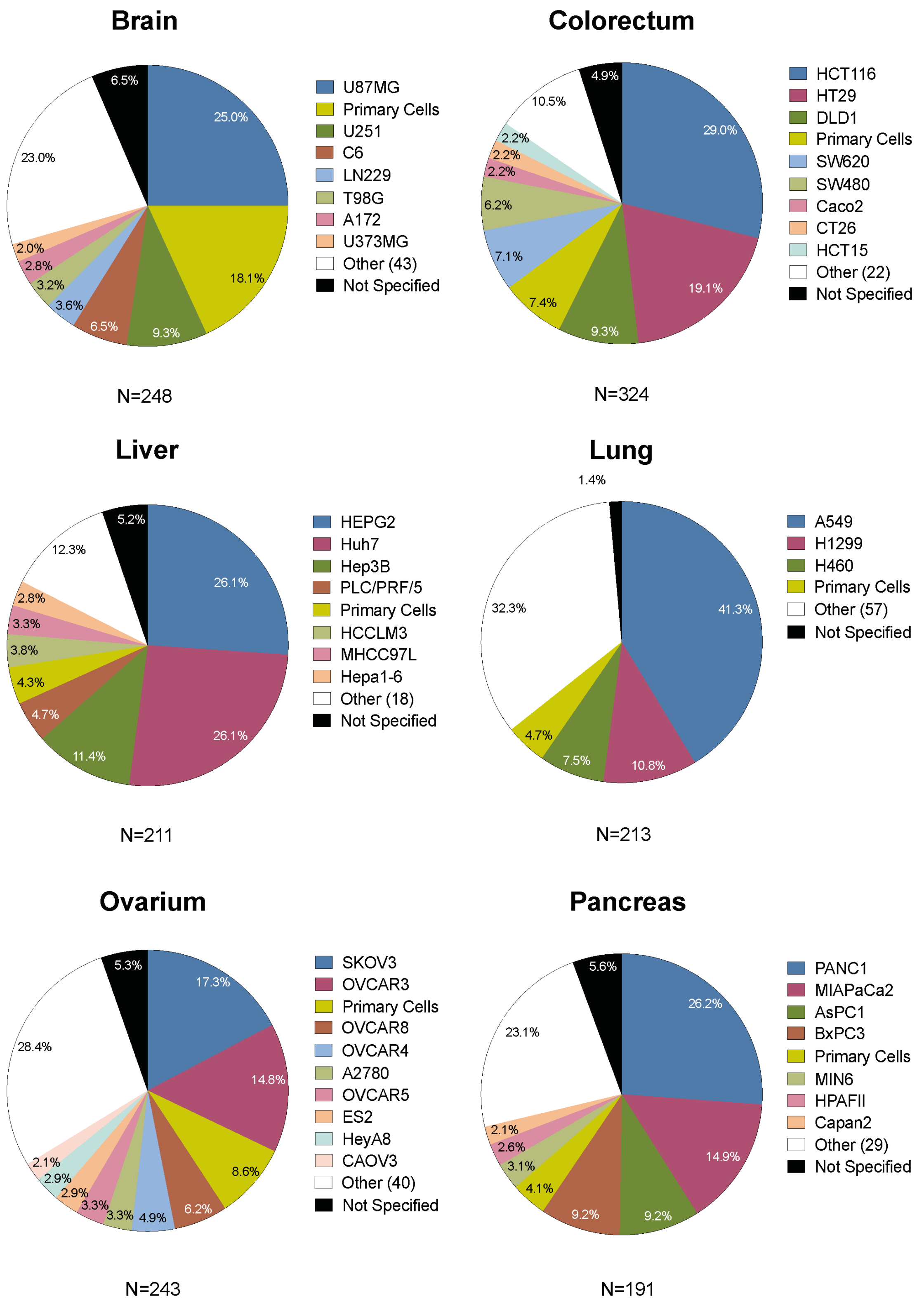

Supplementary Figure 7. Cell line distribution in spheroid experiments from non-breast tumors

Pie chart showing the proportion of cell lines used to prepare spheroids from brain, colorectum, liver, lung, pancreas and ovarium cancer.

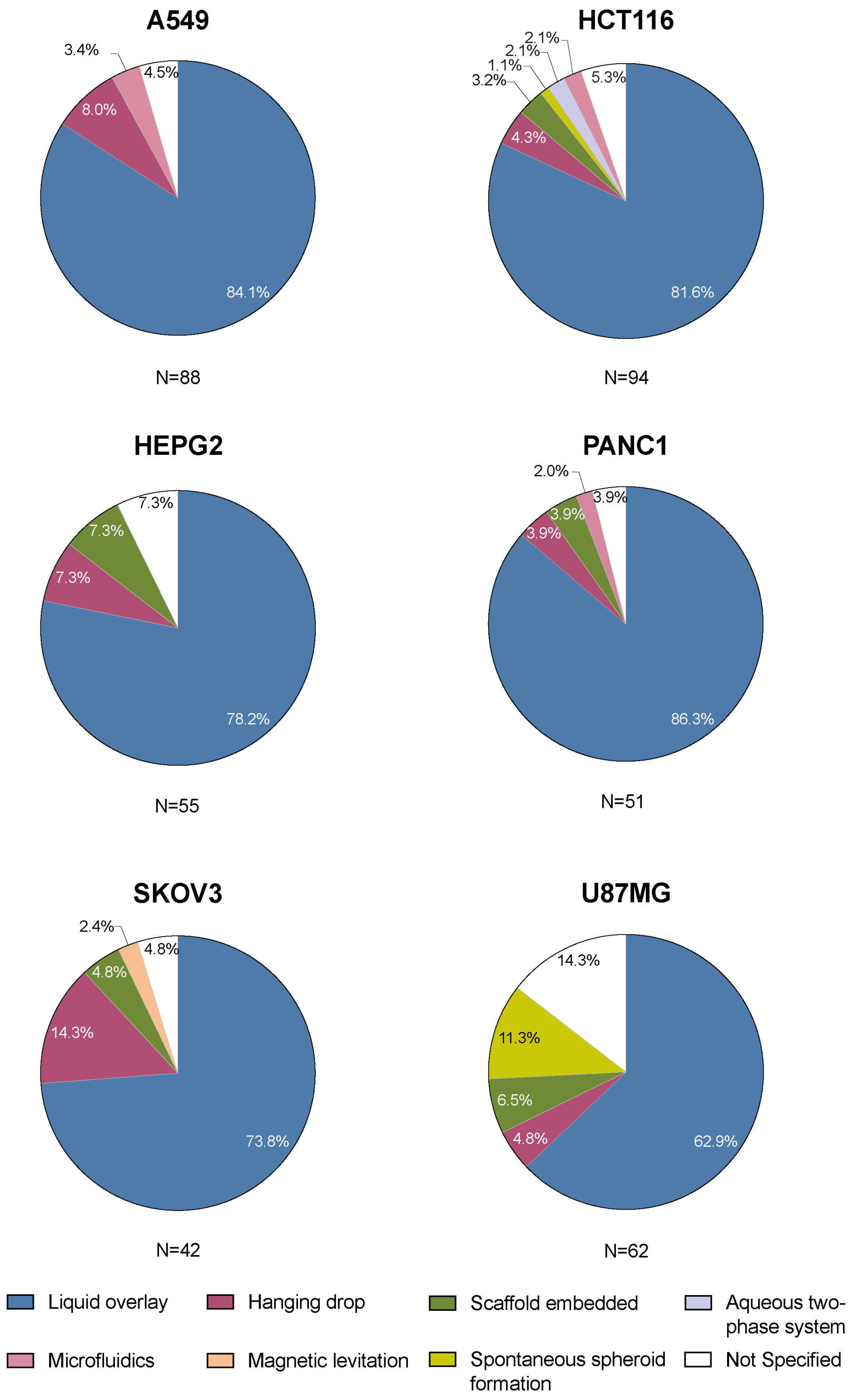

Supplementary Figure 8. Distribution of the applied spheroid formation method to prepare spheroids of the most reported cell line from non-breast tumors

Pie chart visualizing the proportion of formation methods used to prepare A549 (lung), HCT116 (colorectal), HEPG2 (liver), PANC1 (pancreas), SKOV3 (ovarium) and U87MG (brain).

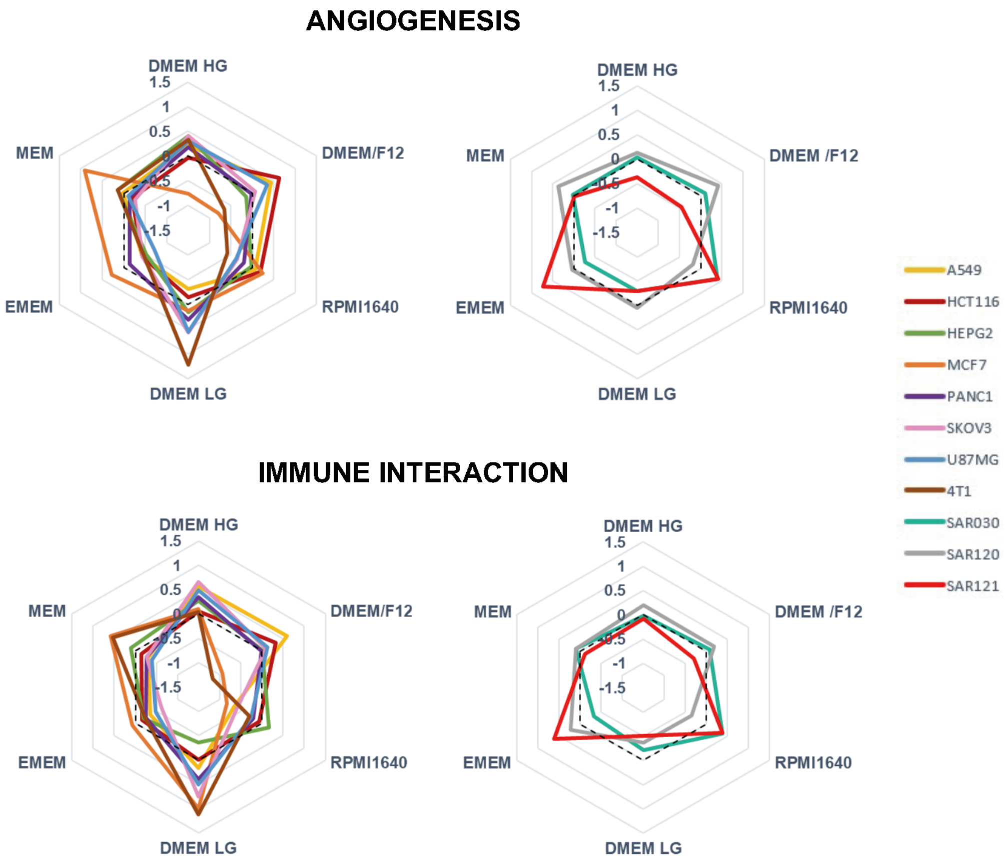

Supplementary Figure 9. Angiogenesis and immune signature metric in spheroid supernatants

Spider plots of angiogenic (upper panel) and immune (lower panel) signature Z-score metrics from supernatants of spheroids of color-coded cell cultures. Axes represent specific medium type. A higher Z-score means a higher metric value. Spider plots on the left indicate established cell lines; on the right early passage patient-derived cultures.

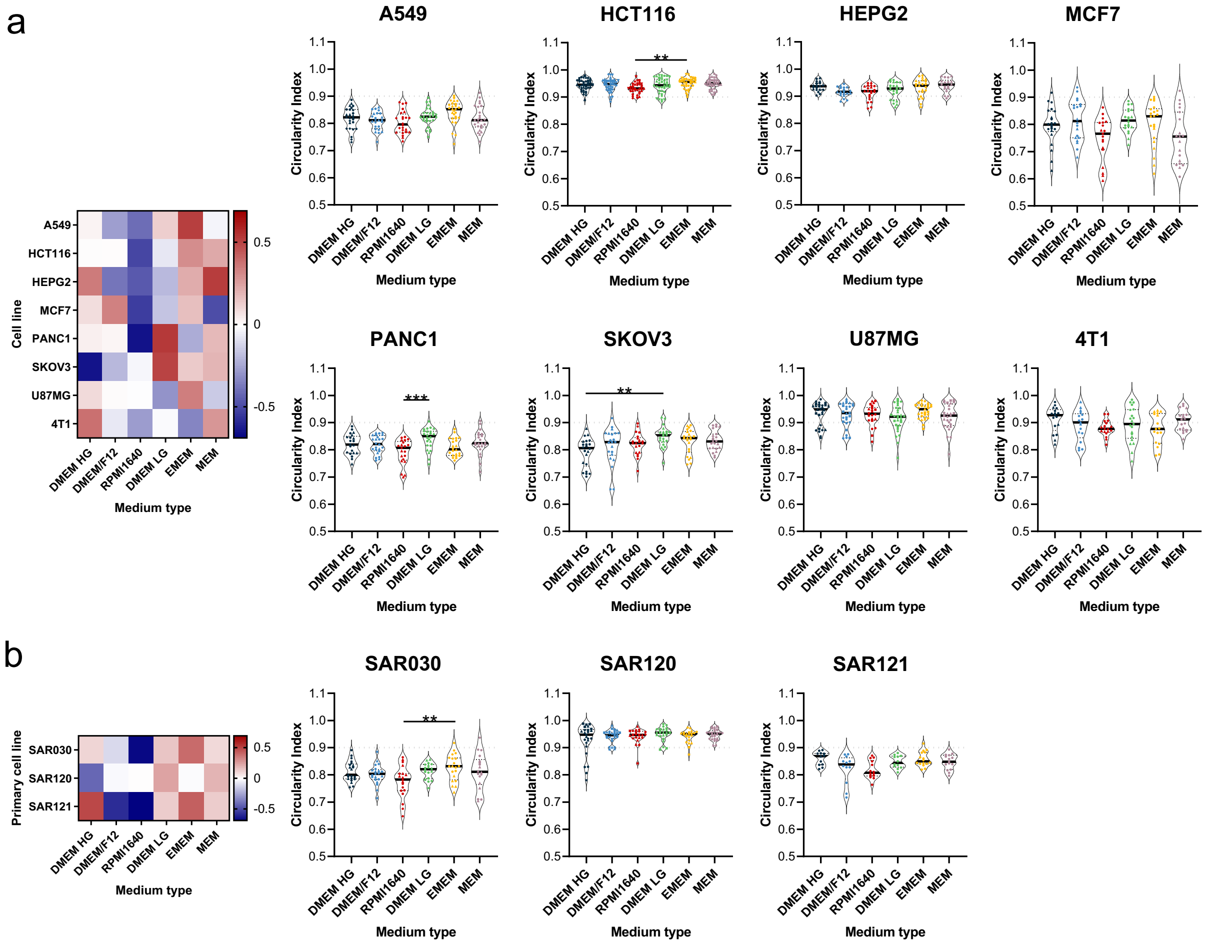

Supplementary Figure 10. Quantitative presentation of the impact of heterogeneity in culture medium on circularity of spheroids

Left, Z-score heatmaps and right, violin plots presenting the impact of six different media types on spheroid circularity in (a) 8 established cell lines and (b) 3 early passage, patient-derived sarcoma cultures. Biological replicates are indicated by a different symbol (N≥3); each symbol is a technical replicate (n=8). Y-axis represents the circularity index. Horizontal bar indicates median. Statistical significance between the groups was determined with a one-way ANOVA and Tukey's multiple comparison test. **p<0.01, ***p<0.001, ****p<0.0001. Colors in violin plots present media type; media types are ranked from higher nutrient (left) to lower nutrient (right) richness.

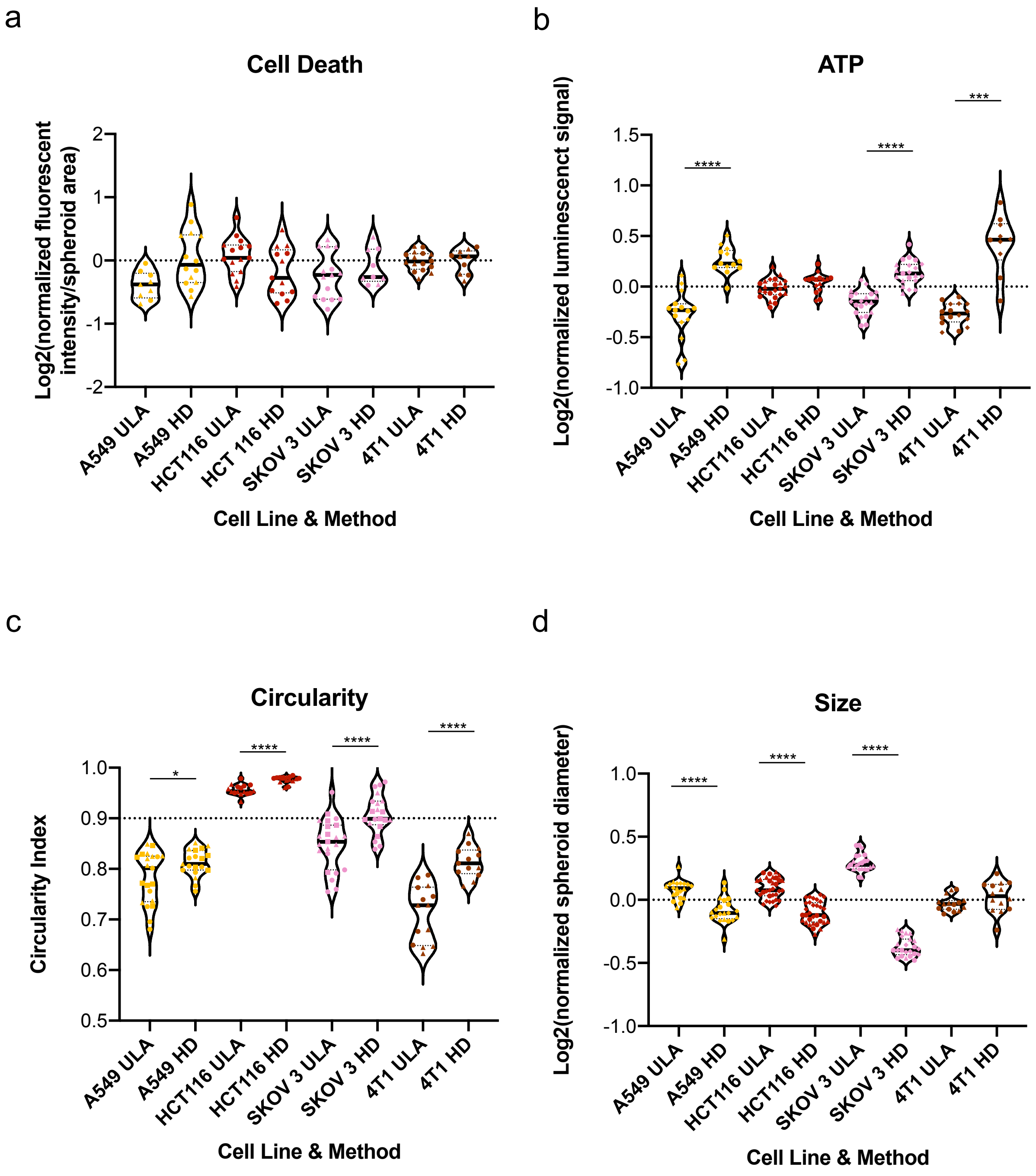

Supplementary Figure 11. Quantitative presentation of the impact of formation method on spheroid metrics

Violin plots presenting the impact of two different spheroid formation methods on (a) cell death, (b) ATP content, (c) circularity and (d) size in spheroids of A549, HCT116, SKOV3 and 4T1 cells. Biological replicates are indicated by a different symbol (N≥2); each symbol is a technical replicate (n=8). Horizontal bar indicates median. Statistical significance between the groups was determined with a one-way ANOVA and Tukey's multiple comparison test. **p<0.01, ***p<0.001, ****p<0.0001.

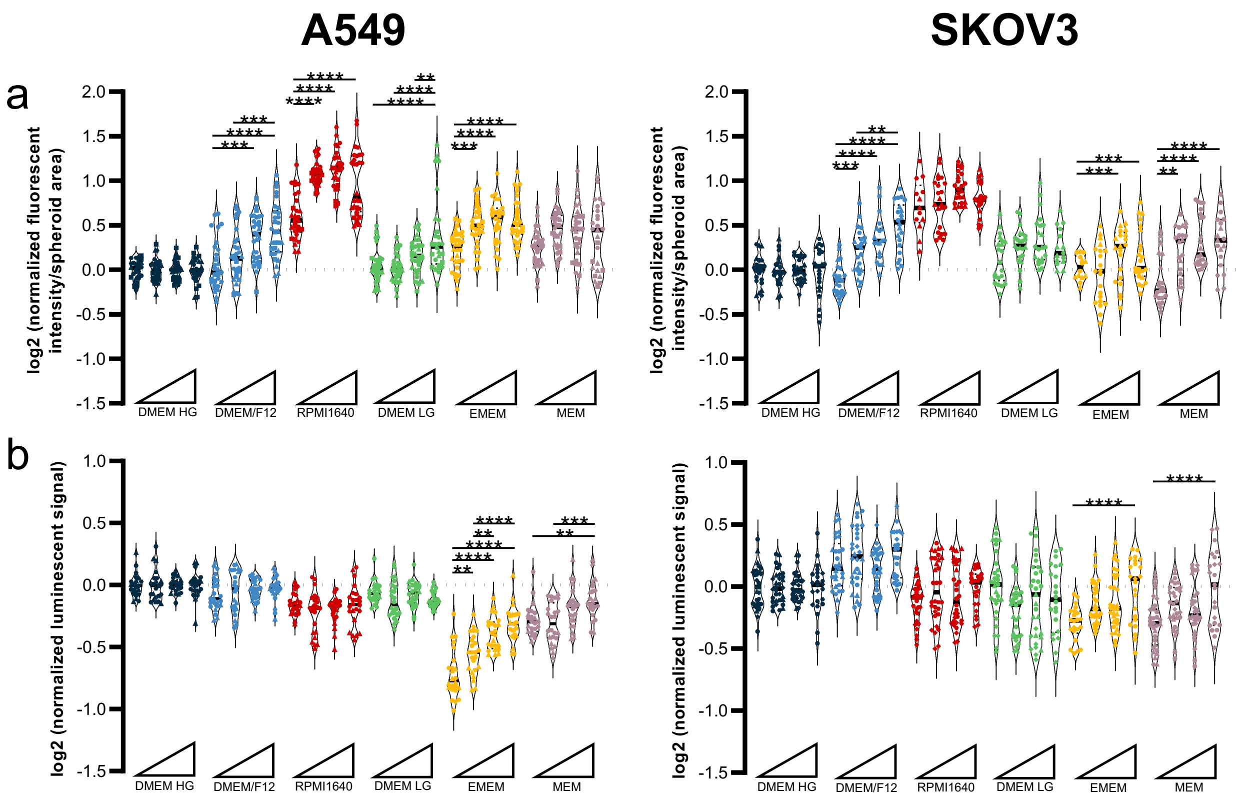

Supplementary Figure 12. Quantitative presentation of the impact of size and different media types on spheroid metrics

Violin plots presenting the impact of size on (a) cell death and (b) ATP content in spheroids from A549 and SKOV3, cultured in six different media types. Biological replicates are indicated by a different symbol (N≥3); each symbol is a technical replicate (n=8). Triangles at X-axis represents increasing seeding cell number and consequently increasing spheroid size. Y-axis represents log2-transformed data, all media types are normalized to DMEM HG. Horizontal bar indicates median. Statistical significance between the groups was determined with a one-way ANOVA and Tukey's multiple comparison test. **p<0.01, ***p<0.001, ****p<0.0001. For size estimates see Supplementary Table 6. Colors in violin plots present media type; media types are ranked from higher nutrient (left) to lower nutrient (right) richness.

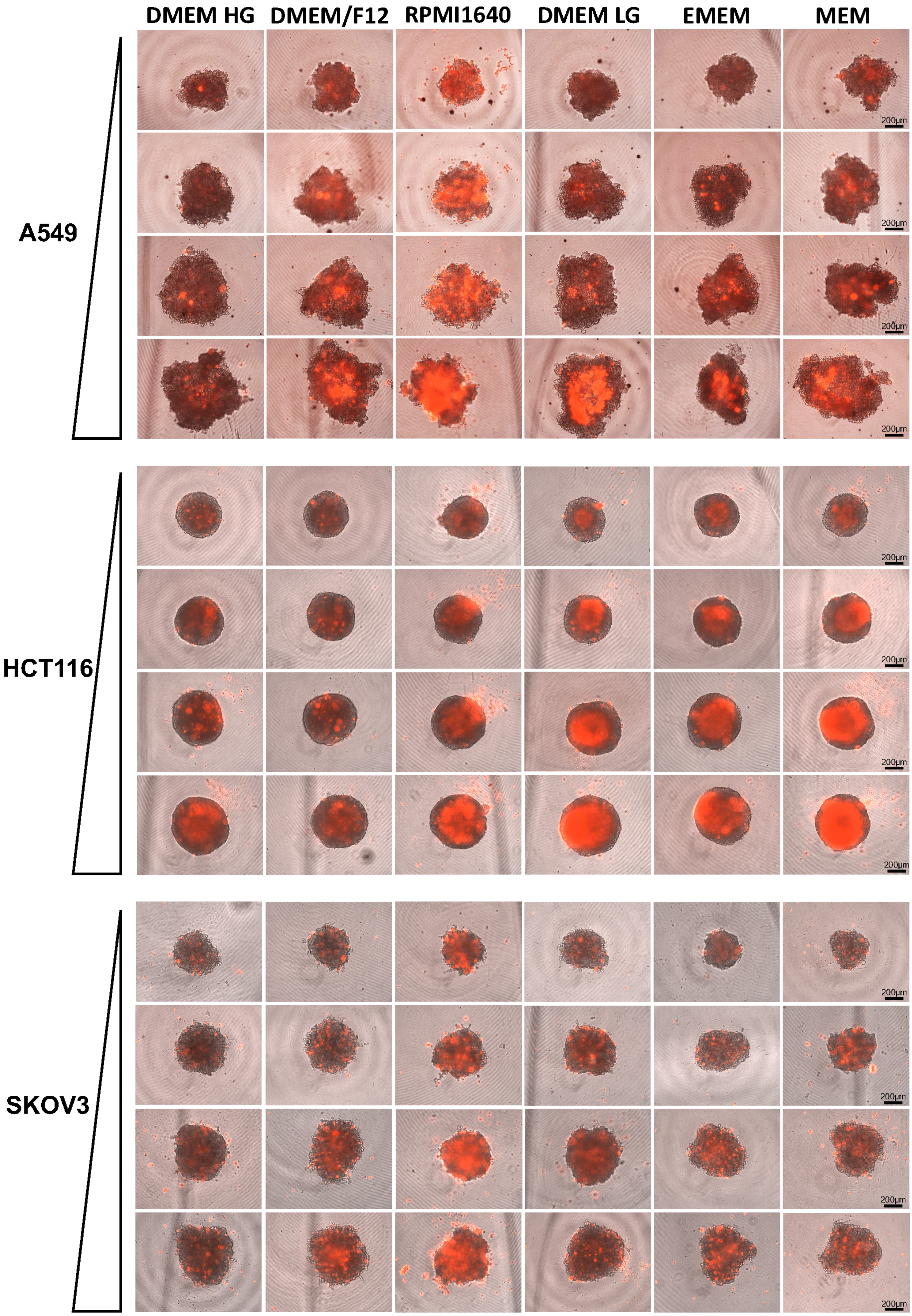
DMEM HG
DMEM/F12
RPMI1640
DMEM LG
EMEM
MEM
A549
HCT116
SKOV3
200μm

Supplementary Figure 13. Image presentation of cell death in differently sized spheroids cultured in six different media types

Representative microscopy images show ethidium homodimer I stain (red if cell is dead) of differently sized spheroids (indicated by triangle left of the images) of A549, HCT116 and SKOV3 cell lines cultured in different media types (indicated in top) (scale bars 200µm). Each experiment was repeated independently at least 3 times with 8 technical replicates per experiment, with similar results. Intense staining in the spheroid center is indicative of necrotic core. For size estimates see Supplementary Table 6. Media types are ranked from higher nutrient (left) to lower nutrient (right) richness.

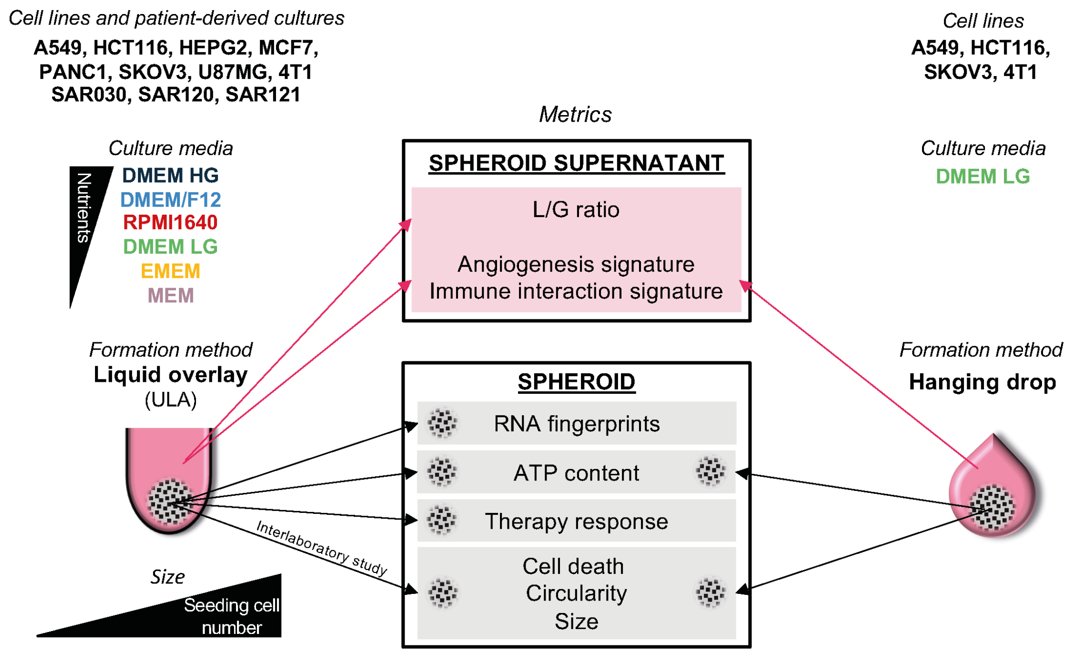

Supplementary Figure 14. Schematic of the empirical setup using variations in the MISpheroID string parameters (cell line, culture medium, formation method and size) evaluated on different spheroid metrics.

Supplementary Tables

Type of cancer	N^{0} of PubMed publications (2010-2019)
Breast	1010
Colorectum	589
Brain	584
Lung	497
Ovarium	409
Liver	380
Pancreas	313

Supplementary Table 1. Spheroid-related publications between 2010-2019, per tissue type.

Supplementary Table 2

**1. Study**	
Study number	Specify
Authors	Specify
Article title	Specify
Journal name	Specify
Journal impact factor from year of publication	Specify
Publication date	Specify
Year of publication	Specify
PMID	Specify
**2. Spheroid experiment setup**	
Experiment number	Specify
Terminology: ***drop-down list***	-Select-
**2.1. Cells**	
Cell line	
Origin confirmation (e.g. STR profile)	Yes or NS
Mycoplasma test	Yes or NS
Coculture in spheroid	Yes or No
If coculture is 'yes': Cell type ***(drop-down list)***	-Select-
If coculture is 'yes': Cell line	Specify
Triculture in spheroid	Yes or No
If triculture is 'yes': Cell type ***(drop-down list)***	-Select-
If triculture is 'yes': Cell line	Specify
**2.2. Culture medium**	Specify
Serum concentration	Specify
Glucose concentration	Specify
Antibiotics	Specify
Supplement: albumin (%)	Specify
Supplement: Glutamine	Yes or No
Supplement: Amino acid solution	Yes or No
Supplement: Growth factors	Yes or No
Supplement: Viscosity enhancer	Specify
If viscosity enhancer is 'yes': %	Specify
Supplement: B27 [1,2]	Yes or No
Supplement: Heparin [1]	Yes or No
Supplement: Insulin [1,2]	Yes or No
Supplement: bFGF [1]	Yes or No
Supplement: EGF [1,2]	Yes or No
Supplement: Corticosteroids [2]	Yes or No
Supplement: Other 1	Specify
Supplement: Other 2	Specify
Supplement: Other 3	Specify
Culture media change (days)	Specify
**2.3. Method of spheroid formation *(drop-down list)***	-Select-
If method is 'scaffold': type of scaffold	Specify
If method is 'liquid overlay': subtype ***(drop-down list)***	-Select-
If method is 'liquid overlay': plate coating ***(drop-down list)***	-Select-
If method is 'liquid overlay': plate size ***(drop-down list)***	-Select-
If method is 'liquid overlay': well bottom shape ***(drop-down list)***	-Select-
Centrifugation step	Yes or No
Spheroid formation time (hours)	Specify
**2.4. Setup**	
Cell number (per well or ml)	Specify

Cell number per spheroid	Specify
Maximum spheroid passage	Specify
Maximum follow-up time (hours)	Specify
Maximum Number of spheroid passages	Specify
**3. Spheroid Characterization**	
**3.1. Visual Characterization**	
Diameter	Specify
Area	Specify
Volume	Specify
Maximum diameter (in size follow-up)	Specify
Shape assessment (e.g. circularity)	Specify
Compactness	Specify
Microscopy: light microscope	Yes or No
Microscopy: phase-contrast microscope	Yes or No
Microscopy: fluorescent microscope	Yes or No
Microscopy: confocal microscope	Yes or No
Microscopy: electron microscope	Yes or No
Microscopy: other	Specify
Fluorescent staining (e.g. GFP, cell tracker)	Specify
Fluorescent analysis: live/dead	Yes or No
Fluorescent analysis: immunofluorescence	Yes or No
Fluorescent analysis: nucleus	Yes or No
Fluorescent analysis: cytoskeleton	Yes or No
Fluorescent analysis: extracellular matrix	Yes or No
Fluorescent analysis: stem cell	Yes or No
Fluorescent drugs: ***(drop-down list)***	-Select-
Fluorescent analysis: other	Specify
Bioluminescence	Yes or No
Histology	Yes or No
IHC: proliferation	Yes or No
IHC: apoptosis	Yes or No
IHC: pluripotency	Yes or No
IHC: hypoxia	Yes or No
IHC: ECM	Yes or No
IHC: Other	Specify
Real time imaging (e.g. video, Incucyte®, Opera®) ***(drop-down list)***	-Select-
**3.2. Non-visual characterization**	
Cell number assessment ***(drop-down list)***	-Select-
Protein analysis	Yes or No
RNA analysis	Yes or No
DNA analysis	Yes or No
Metabolite analysis	Yes or No
Glycan analysis	Yes or No
Lipid analysis	Yes or No
**4. Application *(drop-down list)***	-Select-
Drug treatment	Yes or No
Drug concentration provided	Yes or No
If drug treatment is 'yes': IC50 determined	Yes or No
If IC50 determined is 'yes': method ***drop-down list)***	-Select-
Moment of drug treatment ***(drop-down list)***	-Select-
Stem cell research	Yes or No
Functional assessment: in vitro	Yes or No
Functional assessment: in vivo	Yes or No

Supplementary resources

1. Dontu, G. *et al.* In vitro propagation and transcriptional profiling of human mammary stem/progenitor cells. *Genes Dev.* **17**, 1253–1270 (2003).

2. Grimshaw, M. J. *et al.* Mammosphere culture of metastatic breast cancer cells enriches for tumorigenic breast cancer cells. *Breast Cancer Res.* **10**, 1–10 (2008).

Supplementary Table 2 (in EXCEL FILE). Checklist of 98 reporting parameters concerning experiment identification, spheroid setup, spheroid characterization and application.

Supplementary Table 3

	Cell Line Name	Synonyms
Brain	A-172	**A172**; A 172; A-172 MG; A-172MG
	**C6**	C-6; C 6; RGC-6; RGC6; RGc6
	LN-229	LN 229; **LN229**; LNT-229
	**T98G**	T 98 G; T-98G; T98 G; T98-G
	U-251MG	U-251 MG; U-251-MG; U-251_MG; U251-MG; U251MG; U-251; **U251**; U251n; U251N; 251 MG; 251MG
	U-373MG ATCC	U-373MG; U373 MG; U-373-MG; U-373 MG; U373-MG; **U373MG**; U373; 373 MG; 373MG
	U87-MG ATCC	U-87MG; U-87 MG; U87 MG; U-87-MG; U87-MG; **U87MG**; U-87; U87; 87 MG; 87MG
Breast	**4T1**	4T1-A
	BT-474	Bt-474; **BT474**
	MCF-7	MCF 7; MCF.7; **MCF7**; Michigan Cancer Foundation-7; ssMCF-7; ssMCF7; MCF7/WT; IBMF-7; MCF7-CTRL
	MDA-MB-231	MDA_MB_231; MDA-MB 231; MDA.MB.231; MDA MB 231; MDA MB231; MDA Mb231; MDA-MB231; MDAMB-231; **MDAMB231**; MDA-231; MDA231; MDA231-BRE; MB231; MD Anderson-Metastatic Breast-231
	MDA-MB-468	MDA-MB 468; MDA-MB468; **MDAMB468**; MDA-468; MDA468; MB468; MD Anderson-Metastatic Breast-468
	SK-BR-3	SK-Br-3; Sk-Br-3; SK BR 03; SKBR-3; SKBr-3; SK-BR3; SKBr3; SkBr3; **SKBR3**
	SUM149PT	SUM-149PT; SUM 149PT; SUM149-PT; **SUM149**; SUM-149; SUM 149; 149 PT; 149PT; BrCL12
	SUM159PT	SUM-159-PT; SUM-159PT; SUM 159PT; SUM-159; SUM 159; **SUM159**; 159 PT; 159PT
	T-47D	T-47-D; T47-D; T47D:A; **T47D**
Colorectu	Caco-2	CaCo-2; CACO-2; Caco 2; CACO 2; CACO2; CaCo2; CaCO2; **Caco2**; Caco-2/ATCC; Caco-II
	**CT26**	CT-26; CT 26; CT-26 WT
	DLD-1	DLD 1; **DLD1**; CoCL3
	HCT 116	HCT-116; HCT.116; HCT_116; **HCT116**; CoCL2
	HCT 15	HCT-15; HCT.15; **HCT15**
	HT-29	HT 29; **HT29**
	**SW480**	SW-480; SW 480; SW480E
	**SW620**	SW-620; SW 620; SW.620
Liver	**HCCLM3**	LM3; MHCC-LM3; MHCCLM3
	Hep 3B2.1-7	Hep 3B2_1-7; HEP3B217; Hep 3B2; HEP-3B2; HEP3B2; Hep-3B; HEP-3B; Hep 3B; **Hep3B**; HEP3B
	Hepa 1-6	HEPA 1-6; Hepa-1-6; **Hepa1-6**
	Hep-G2	HEP-G2; Hep G2; HEP G2; HepG2; **HEPG2**
	Huh-7	HuH-7; HUH-7; HuH7; **Huh7**; HUH7; HUH7.0; JTC-39; Japanese Tissue Culture-39
	MHCC97-L	MHCC 97-L; **MHCC97L**
	**PLC/PRF/5**	PLC-PRF-5; PLC PRF 5; PLC/PRF5; PLCPRF5; PLC-8024; PLC8024; PLC; Alexander cells; Alexander; Primary Liver Carcinoma/Poliomyelitis Research Foundation/5
Lung	A549	A 549; **A549**; NCI-A549; A549/ATCC; A549 ATCC; A549ATCC; hA549
	NCI-H1299	**H1299**; H-1299; NCIH1299
	NCI-H460	NCI.H460; **H460**; H-460; NCIH460; NCI-HUT-460; NCI-460
Ovarium	**A2780**	A-2780; 2780; A2780S
	Caov-3	CaOv-3; CaOV-3; CAOV-3; **CAOV3**; CaOV3; CaOv3; Caov3; CA-OV-3
	**ES2**	ES-2
	HEY A8	HEY-A8; Hey-A8; Hey A8; HEYA8; **HeyA8**
	OVCAR-3	Ovcar-3; OVCAR 3; OVCAR.3; NIH:OVCAR-3; NIH:Ovcar-3; NIH:OVCAR3; NIH-OVCAR-3; NIHOVCAR3; **OVCAR3**; Ovcar3
	OVCAR-4	OVCAR 4; NIH:OVCAR-4; NIH:OVCAR4; OVCAR.4; **OVCAR4**; Ovcar4
	OVCAR-5	OVCAR 5; NIH:OVCAR-5; OVCAR.5; **OVCAR5**; Ovcar5; OVCA5
	OVCAR-8	OVCAR 8; NIH:OVCAR-8; **OVCAR8**; Ovcar8; OVCAR.8; OVCA8
	SK-OV-3	SKOV-3; SK-OV3; SK.OV.3; **SKOV3;** Skov3; SKO3
Pancreas	AsPC-1	AsPc-1; Aspc-1; ASPC-1; As-PC1; ASPC1; **AsPC1**; Aspc1; AsPc1
	BxPC-3	BxPc-3; BXPC-3; Bx-PC3; BXPC3; **BxPC3**; BxPc3; Biopsy xenograft of Pancreatic Carcinoma line-3
	Capan-2	CaPan-2; CAPAN-2; Capan 2; CAPAN 2; **Capan2**; CAPAN2
	HPAF-II	HPAF II; **HPAFII**; HPAF-2; HPAF2; HPAF/CD18; CD18/HPAF; HPAF-II/CD18; CD-18; CD18; CD 18
	MIA PaCa-2	MIA-PaCa-2; MIA-PACA-2; MIA-Pa-Ca-2; MIA Paca2; MIA PaCa2; MiaPaCa-2; MIAPACA-2; MiaPaca.2; **MiaPaCa2**; Miapaca2; MIAPaCa2; MIAPACA2; Mia PACA 2; MIAPaCa-2; PaCa2
	**MIN6**	Min6; MIN-6; Mouse INsulinoma 6
	PANC-1	Panc-1; PANC.1; Panc 1; PanC1; Panc1; **PANC1**; Panc-1-P

Supplementary Table 3: Original cell line names and synonyms (according to cellosaurus: https://web.expasy.org/cellosaurus/). In this manuscript the simplest unambiguous cell line name notation (marked in bold) was applied.

Supplementary Table 4

	Tukey's multiple comparisons test	Mean Difference	Significance
A549	DMEM HG vs. DMEM/F12	0.4155	ns
	**DMEM HG vs. RPMI1640**	**-1.472**	****
	**DMEM HG vs. DMEM LG**	**-0.9176**	****
	**DMEM HG vs. EMEM**	**-1.434**	****
	**DMEM HG vs. MEM**	**-1.480**	****
	**DMEM/F12 vs. RPMI1640**	**-1.887**	****
	**DMEM/F12 vs. DMEM LG**	**-1.333**	****
	**DMEM/F12 vs. EMEM**	**-1.850**	****
	**DMEM/F12 vs. MEM**	**-1.896**	****
	**RPMI1640 vs. DMEM LG**	**0.5541**	**
	RPMI1640 vs. EMEM	0.03772	ns
	RPMI1640 vs. MEM	-0.008491	ns
	**DMEM LG vs. EMEM**	**-0.5164**	*
	**DMEM LG vs. MEM**	**-0.5626**	**
	EMEM vs. MEM	-0.04621	ns
HCT116	DMEM HG vs. DMEM/F12	-0.2085	ns
	DMEM HG vs. RPMI1640	0.4856	ns
	DMEM HG vs. DMEM LG	0.3096	ns
	DMEM HG vs. EMEM	0.4313	ns
	**DMEM HG vs. MEM**	**0.8749**	*
	DMEM/F12 vs. RPMI1640	0.6941	ns
	DMEM/F12 vs. DMEM LG	0.5181	ns
	DMEM/F12 vs. EMEM	0.6397	ns
	**DMEM/F12 vs. MEM**	**1.083**	**
	RPMI1640 vs. DMEM LG	-0.1760	ns
	RPMI1640 vs. EMEM	-0.05438	ns
	RPMI1640 vs. MEM	0.3893	ns
	DMEM LG vs. EMEM	0.1216	ns
	DMEM LG vs. MEM	0.5653	ns
	EMEM vs. MEM	0.4437	ns
SKOV3	DMEM HG vs. DMEM/F12	-0.3051	ns
	**DMEM HG vs. RPMI1640**	**-0.8767**	*
	DMEM HG vs. DMEM LG	0.04199	ns
	DMEM HG vs. EMEM	-0.5893	ns
	DMEM HG vs. MEM	0.2130	ns
	DMEM/F12 vs. RPMI1640	-0.5715	ns
	DMEM/F12 vs. DMEM LG	0.3471	ns
	DMEM/F12 vs. EMEM	-0.2841	ns
	DMEM/F12 vs. MEM	0.5181	ns
	**RPMI1640 vs. DMEM LG**	**0.9186**	**
	RPMI1640 vs. EMEM	0.2874	ns
	**RPMI1640 vs. MEM**	**1.090**	***
	DMEM LG vs. EMEM	-0.6312	ns
	DMEM LG vs. MEM	0.1710	ns
	**EMEM vs. MEM**	**0.8023**	*
U87MG	**DMEM HG vs. DMEM/F12**	**1.814**	****
	**DMEM HG vs. RPMI1640**	**1.679**	****
	**DMEM HG vs. DMEM LG**	**1.406**	****
	**DMEM HG vs. EMEM**	**2.454**	****
	**DMEM HG vs. MEM**	**1.744**	****
	DMEM/F12 vs. RPMI1640	-0.1356	ns
	DMEM/F12 vs. DMEM LG	-0.4084	ns
	**DMEM/F12 vs. EMEM**	**0.6400**	*
	DMEM/F12 vs. MEM	-0.07093	ns
	RPMI1640 vs. DMEM LG	-0.2727	ns
	**RPMI1640 vs. EMEM**	**0.7756**	**
	RPMI1640 vs. MEM	0.06470	ns
	**DMEM LG vs. EMEM**	**1.048**	****
	DMEM LG vs. MEM	0.3374	ns
	**EMEM vs. MEM**	**-0.7109**	*

Supplementary Table 4. Overall significance of all cancer hallmarks genes differentially expressed between indicated two media types in A549, HCT116, SKOV3 and U87MG.

Supplementary Table 5

Angiogenesis	Immune interaction
Eotaxin	Eotaxin
Eotaxin-2	Eotaxin-2
Fractalkine	Fractalkine
IL-1α	IL-1α
IL-1β	IL-1β
IL-6	IL-6
IL-8	IL-8
MCP-1	MCP-1
TNFα	TNFα
EGF	sCD40L
FGF-1	G-CSF
FGF-2	M-CSF
HGF	GM-CSF
PDGF-AA	ENA-78
PDGF-AB/BB	GROα
PLGF	I-309
VEGF-A	IFN-α2
VEGF-C	IFNγ
	IL-1RA
	IL-4
	IL-9
	IL-12p40
	IL-13
	IL-15
	IL-16
	IL-17A
	IL-22
	IL-27
	IL-28A
	IP-10
	KC
	LIF
	MCP-2
	MCP-3
	MIG/CXCL9
	MIP-1α
	MIP-1δ
	RANTES
	SDF-1α+β
	TNFβ

Supplementary Table 5. Overview of evaluated secreted protein signatures in angiogenesis and immune interactions.

Supplementary Table 6

Cell line	Seeding cell number (cells/well)	Culture medium					
		**DMEM HG**	**DMEM/F12**	**RPMI1640**	**DMEM LG**	**EMEM**	**MEM**
		Diameter ± SD (µm)					
**A549**	2000	549 ± 68	528 ± 44	553 ± 42	549 ± 46	480 ± 59	551 ± 56
	4000	683 ± 86	645 ± 50	642 ± 42	662 ± 64	598 ± 48	664 ± 60
	6000	737 ± 78	694 ± 59	706 ± 46	710 ± 62	658 ± 54	699 ± 54
	8000	791 ± 90	747 ± 75	728 ± 52	757 ± 72	697 ± 70	755 ± 71
**HCT116**	500	505 ± 16	522 ± 13	537 ± 20	524 ± 43	539 ± 27	498 ± 29
	1000	563 ± 15	564 ± 16	566 ± 24	596 ± 47	580 ± 24	560 ± 30
	2000	615 ± 16	597 ± 13	621 ± 30	664 ± 40	629 ± 23	618 ± 21
	3000	642 ± 18	617 ± 15	635 ± 24	704 ± 30	643 ± 20	636 ± 20
**HEPG2**	2000	592 ± 19	631 ± 32	583 ± 34	575 ± 29	559 ± 22	540 ± 22
**MCF7**	2000	565 ± 80	518 ± 72	573 ± 67	537 ± 47	562 ± 51	552 ± 53
**PANC1**	2000	688 ± 32	700 ± 42	719 ± 29	730 ± 62	705 ± 16	658 ± 26
**SKOV3**	2000	532 ± 33	522 ± 23	515 ± 62	505 ± 36	477 ± 50	468 ± 35
	4000	599 ± 41	599 ± 24	569 ± 50	588 ± 48	558 ± 53	553 ± 36
	6000	632 ± 31	636 ± 29	611 ± 57	622 ± 40	607 ± 35	590 ± 34
	8000	664 ± 48	664 ± 31	626 ± 56	627 ± 36	631 ± 45	624 ± 36
**U87MG**	2000	593 ± 42	589 ± 41	592 ± 36	570 ± 37	500 ± 36	554 ± 41
**4T1**	2000	408 ± 30	370 ± 23	386 ± 26	441 ± 34	348 ± 23	370 ± 26
**SAR030**	8000	495 ± 21	467 ± 20	510 ± 29	505 ± 39	431 ± 46	457 ± 29
**SAR120**	2000	450 ± 23	455 ± 19	433 ± 17	439 ± 21	396 ± 28	454 ± 25
**SAR121**	2000	366 ± 18	354 ± 19	374 ± 29	381 ± 31	379 ± 38	369 ± 19

Supplementary Table 6. Sizes of spheroids from established cell lines and early passage patient-derived cell cultures evaluated in six different media types.

Supplementary Table 7

	SAR030	SAR120	SAR121
**Gender**	Female	Female	Male
**Age at diagnosis**	71	33	83
**Grade**	High-grade	Grade 1	High-grade
**Sarcoma subtype**	Undifferentiated pleomorphic spindle cell sarcoma, not otherwise specified	Chondrosarcoma	Synovial sarcoma
**Primary tumour / metastasis**	Primary tumour	Primary tumour	Primary tumour
**Tumour location**	Left upper leg	Left femur	Left lower arm
**Tissue sampling for cell-line development**	Resection	Biopsy	Biopsy
**Neo-adjuvant treatment before tissue sampling**	No	No	No

Supplementary Table 7. Source of the early passage patient-derived sarcoma cultures SAR030, SAR120 and SAR121.
