# Supplementary material for: MISpheroID: a knowledgebase and transparency tool for minimum information in spheroid identity
Source: Nat Methods. 2021 Nov 1;18(11):1294–303. doi: 10.1038/s41592-021-01291-4 (PMC8566242; doi:10.1038/s41592-021-01291-4)
Supplement: Supplementary file 2 — Reporting Summary [file 41592_2021_1291_MOESM2_ESM.pdf]

Corresponding author(s): Olivier De Wever

Last updated by author(s): Aug 27, 2021

Reporting Summary

Nature Research wishes to improve the reproducibility of the work that we publish. This form provides structure for consistency and transparency in reporting. For further information on Nature Research policies, see our Editorial Policies and the Editorial Policy Checklist.

Statistics

For all statistical analyses, confirm that the following items are present in the figure legend, table legend, main text, or Methods section.

n/a	Confirmed	
☐	☒	The exact sample size (*n*) for each experimental group/condition, given as a discrete number and unit of measurement
☐	☒	A statement on whether measurements were taken from distinct samples or whether the same sample was measured repeatedly
☐	☒	The statistical test(s) used AND whether they are one- or two-sided<br>*Only common tests should be described solely by name; describe more complex techniques in the Methods section.*
☒	☐	A description of all covariates tested
☐	☒	A description of any assumptions or corrections, such as tests of normality and adjustment for multiple comparisons
☐	☒	A full description of the statistical parameters including central tendency (e.g. means) or other basic estimates (e.g. regression coefficient) AND variation (e.g. standard deviation) or associated estimates of uncertainty (e.g. confidence intervals)
☐	☒	For null hypothesis testing, the test statistic (e.g. *F*, *t*, *r*) with confidence intervals, effect sizes, degrees of freedom and *P* value noted<br>*Give P values as exact values whenever suitable.*
☒	☐	For Bayesian analysis, information on the choice of priors and Markov chain Monte Carlo settings
☒	☐	For hierarchical and complex designs, identification of the appropriate level for tests and full reporting of outcomes
☐	☒	Estimates of effect sizes (e.g. Cohen's *d*, Pearson's *r*), indicating how they were calculated

Our web collection on statistics for biologists contains articles on many of the points above.

Software and code

Policy information about availability of computer code

Data collection	NextSeq 500 software v.4.0.1; Gen5 Data analysis software v3.08.01; AxioVision Rel. 4.8
Data analysis	DESeq2 v1.30.1; GSEA software v4.1.0; PAST4.03; AnaSP v1.4; Excel v.2106; Graphpad Prism v8.4.3; ImageJ v1.52v.

For manuscripts utilizing custom algorithms or software that are central to the research but not yet described in published literature, software must be made available to editors and reviewers. We strongly encourage code deposition in a community repository (e.g. GitHub). See the Nature Research guidelines for submitting code & software for further information.

Data

Policy information about availability of data

All manuscripts must include a data availability statement. This statement should provide the following information, where applicable:

- Accession codes, unique identifiers, or web links for publicly available datasets
- A list of figures that have associated raw data
- A description of any restrictions on data availability

RNA sequencing data generated during the current study will be available in the ArrayExpress database (www.ebi.ac.uk/arrayexpress) under accession number E-MTAB-10862 .
The source data underlying Figure 4 and 5, Extended Figures 4-10, Supplementary Figures 9-13 and Table 6 are provided as a source data file. All literature study data are available through the MISpheroID knowledgebase.

Field-specific reporting

Please select the one below that is the best fit for your research. If you are not sure, read the appropriate sections before making your selection.

☒ Life sciences ☐ Behavioural & social sciences ☐ Ecological, evolutionary & environmental sciences

For a reference copy of the document with all sections, see nature.com/documents/nr-reporting-summary-flat.pdf

Life sciences study design

All studies must disclose on these points even when the disclosure is negative.

Sample size	Dependent on the metric investigated, four (transcriptional variation and ratio of glucose uptake to lactate secretion) to minimally eight (all other) technical replicates are included in each experiment, reproducibility was further demonstrated with multiple biological replicates. Sample size are indicated in the legends of all relevant figures and in the Material&Methods section. Sample sizes were justified a posteriori since differences were highly statistical significant (p-values lower than 0.01 are shown).
Data exclusions	Data points were first excluded manually (e.g. spheroids containing a dust particle when measuring dead signal, spheroids that were lost when pipetting to a white plate for measuring metabolic activity with cellTiter-glo3D, etc.). Afterwards, outliers were calculated via the ROUT method (Q=1%) in the Graphpad Prism software.
Replication	Reproducibility of the experimental findings was verified in at least 3 biological replicates for most spheroid metrics and an interlaboratory study between 7 sites investigates the impact of culture medium type on three spheroid metrics. All attempts at replication were succesful.
Randomization	No randomization was included as this was not relevant to the study design.
Blinding	The study was not blinded since dead stainings were often distinctly observable between groups (dead staining assay) or the results were obtained computationally (all other assays).

Reporting for specific materials, systems and methods

We require information from authors about some types of materials, experimental systems and methods used in many studies. Here, indicate whether each material, system or method listed is relevant to your study. If you are not sure if a list item applies to your research, read the appropriate section before selecting a response.

Materials & experimental systems

n/a	Involved in the study
☒	☐ Antibodies
☐	☒ Eukaryotic cell lines
☒	☐ Palaeontology and archaeology
☒	☐ Animals and other organisms
☒	☐ Human research participants
☒	☐ Clinical data
☒	☐ Dual use research of concern

Methods

n/a	Involved in the study
☒	☐ ChIP-seq
☒	☐ Flow cytometry
☒	☐ MRI-based neuroimaging

Eukaryotic cell lines

Policy information about cell lines

Cell line source(s)	The following cell lines: A549, HCT116, HEPG2, MCF7, PANC1, SKOV3, U87MG and 4T1 were purchased from the American Type Culture Collection. SAR030, SAR120 and SAR121 are primary cell cultures established in the lab.
Authentication	All human cell lines were authenticated using a 21-Marker STR Profile test (Eurofins, Luxembourg).
Mycoplasma contamination	All cell lines were monthly tested for mycoplasma contamination using the Mycoalert Mycoplasma Detection Kit and tested negative.
Commonly misidentified lines (See ICLAC register)	No commonly misidentified cell lines were used in the study.
